# Concrete multi-agent path planning enabling kinodynamically aggressive maneuvers

**DOI:** 10.1038/s44182-026-00083-2

**Published:** 2026-03-14

**Authors:** Keisuke Okumura, Guang Yang, Zhan Gao, Heedo Woo, Amanda Prorok

**Affiliations:** 1 University of Cambridge, Cambridge, UK; 2 National Institute of Advanced Industrial Science and Technology (AIST), Tokyo, Japan

**Keywords:** Engineering, Mathematics and computing

## Abstract

Coordinated trajectory planning is essential for multi-robot applications, ranging from factory automation to entertainment. The main challenge is providing long-term coordination guarantees, such as freedom from collisions, deadlocks, and livelocks, as well as kinodynamic agility, especially in densely populated environments. Although continuous optimization provides agility, it is computationally expensive. In contrast, discrete search is scalable but lacks physical realism for robot execution. This study introduces concrete planning, a hybrid approach that captures real-world continuous dynamics while maintaining scalable guaranteed planning via discrete search. We integrate recent advances in robot dynamics learning, optimal control, and anytime complete planning into a modular framework. The framework is deployed with 40 robots, including 20 aerial, 8 ground, and 12 obstacle robots, operating in a compact laboratory space. Despite the dense and time-varying setup, the robots achieve consecutive navigation missions on-demand, while executing aggressive maneuvers that substantially reduce task completion time.

## Introduction

The ability to navigate multiple robots in a shared workspace is a keystone technology for every large-scale automated system. This is evident in a range of applications such as factory automation^1,2^, traffic management^3,4^, manufacturing^5,6^, and delivery services^7^, to name just a few. In essence, all tasks related to multi-robot navigation can be distilled to a one-shot problem of guiding each robot to its intended destination in a shared environment, given their initial configuration. The concept of coordination is then further specified as the absence of inter-robot collisions, deadlocks, or livelocks that could impede mission progress, while optimizing the resulting motion trajectories to satisfy user-defined objectives, such as minimizing mission completion time.

Coordinated navigation presents unique robotic challenges not found in single-robot systems, such as cooperative perception and reliable inter-robot communication^8^. Our focus is on the critical challenge of long-term trajectory planning to achieve coordinated multi-robot behavior. This entails a computational challenge that increases exponentially with the number of robots, lying at the root of the difficulty in achieving (i) *scalable* systems consisting of large numbers of robots; (ii) *reliable* systems that can guarantee safe and efficient coordination; (iii) systems permitting *kinodynamically aggressive* maneuvers to exploit hardware potential and maximize performance; and (iv) *mission-live* systems capable of swiftly adapting to time-varying configurations to ensure uninterrupted mission continuity during deployment.

At one end of the solution spectrum are *decoupled* trajectory planning methods. Often decentralized (though sometimes centralized), they are popular due to their reduced computational overhead, which is achieved by dividing the joint coordination into individual robot subproblems^9–17^. While decoupled strategies provide practical solutions, particularly in resource-constrained or partially observable scenarios, they come at the cost of sub-optimal behavior, inapplicability to scenarios requiring tight coordination, and the loss of long-term guarantees such as collision- and deadlock-freeness. These assurances and an understanding of coordination beyond its mere emergence are necessary for the lifelong infrastructure systems that will be integral to our future. Therefore, practical demands also require us to develop *coupled* planning techniques with guarantees that simultaneously offer scalability, reliability, kinodynamic agility, and mission-liveness.

The computational cost of coupled planning, which operates in the joint configuration space of all robots, is primarily governed by the degree of robot state discretization, a factor that also influences the achievable kinodynamic agility. At one extreme, using a discrete spatiotemporal representation leads to the multi-agent pathfinding problem (MAPF)^18^, typically solved as graph-based combinatorial search. MAPF has undergone substantial development since the 2010s^19–21^, with recent scalable discrete search techniques capable of managing on the order of 10^3^ to 10^4^ agents^22–25^ while maintaining mission-liveness. However, its fundamental assumption of a simplified grid-world hinders direct adaptation to real-world robots with kinodynamic constraints. Conversely, numerous studies conceptualize multi-robot navigation as a *numerical optimization process* within continuous state space representations^12,14,16,26–31^. This direction enables the synthesis of smooth and aggressive maneuvers based on high-fidelity dynamics but at a greater computational expense. As a result, such methods offering coordination guarantees often remain limited to just several agents^26–30^ to ensure real-time responsiveness.

**Fig. 1:**
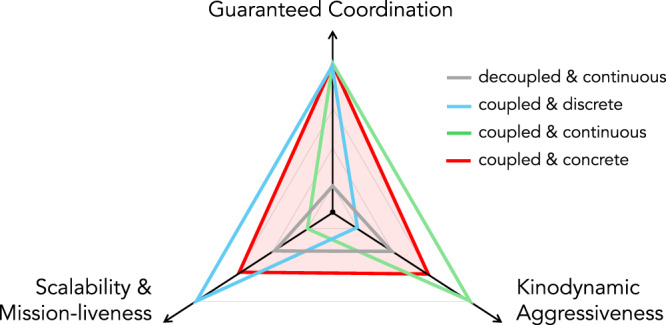
Conceptual positioning of multi-agent trajectory planning strategies. This schematic illustrates how a *coupled and concrete* approach, when feasible, optimizes the balance between coordination guarantees and performance. While this diagram represents a qualitative framework, the trade-offs depicted are empirically validated by the experimental results (e.g., Figs. 3 and 4).

Each extreme of the planning discretization spectrum offers specific benefits alongside distinct drawbacks. This disparity motivates the development of a nuanced state representation that permits scalable discrete search while effectively capturing the robot’s continuous-domain motion. We term this planning paradigm “concrete,” a name derived from its hybrid continuous and discrete state representation, inspired by the seminal computer science textbook^32^. Beyond the inherent coordination guarantees of its coupled formulation, concrete planning aims to offer improved scalability and real-time responsiveness compared to fully continuous methods, due to its use of discrete search. Simultaneously, it enables kinodynamically aggressive motion by maintaining awareness of the system’s low-level dynamics (Fig. 1).

This study crystallizes the notion of *concrete* multi-agent path planning. Given a spatiotemporal motion model, a solution is a sequence of coordinated waypoints for each robot, directly and independently executable with an individual robot controller that converts waypoints into dynamically feasible state trajectories. In order to solve this planning problem, we develop a theoretically complete and optimal algorithm termed Tree-LaCAM, which is based on a leading scalable MAPF algorithm, i.e., LaCAM and versions thereof^24,33,34^. Tree-LaCAM finds cost-minimizing optimal solutions in finite time if a solution exists, and otherwise, reports the nonexistence of a solution. In practice, Tree-LaCAM is an anytime algorithm, allowing it to quickly find feasible solutions and gradually refine them towards optimal solutions as time progresses.

**Fig. 2:**
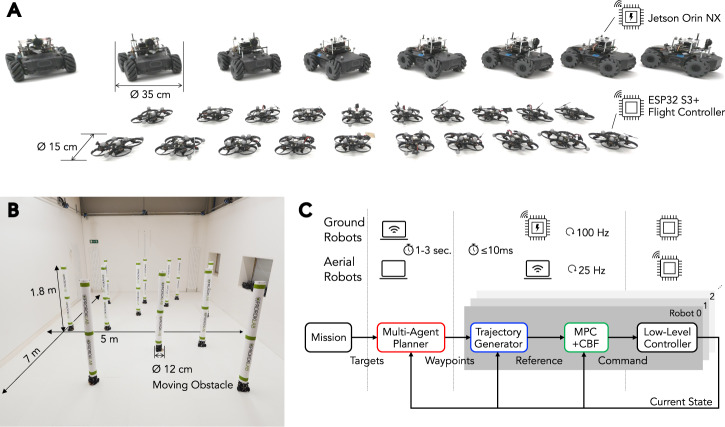
System overview. **A** Our robot fleet of 8 ground and 20 aerial robots. The ground robots are based on the Cambridge RoboMaster^67^, while the aerial robots are a newly built platform^68^. **B** Indoor experimental workspace. The motion capture system tracks objects within 7.0 × 5.0 × 1.8 m^3^ space. The snapshot includes 12 TurtleBots acting as moving obstacles. **C** Planning and control pipeline. Given a multi-robot navigation mission, an offboard coupled multi-agent planner assigns coordinated waypoint sequences to each robot. Reference trajectories are then generated within a per-robot controller and tracked by an MPC controller with CBF reactive collision avoidance. The individual robot controllers operate either onboard or offboard the robot, depending on the computational capabilities of the platform.

**Fig. 3:**
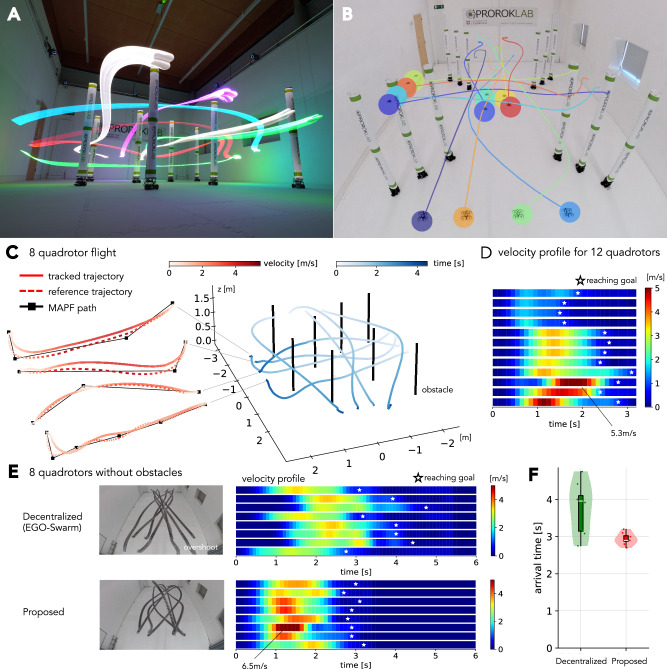
Aggressive multi-quadrotor navigation. **A** Long-exposure photo of an eight-quadrotor flight mission. Four quadrotors flew from back to front, and the rest from right to left. **B** Trajectory overlay for a twelve-quadrotor mission, projected using recorded data. Their initial positions are shown in the image. **C** Framework visualization with the eight-quadrotor mission. The illustration includes waypoints generated by MAPF, reference trajectories given these waypoints, and the actual trajectories tracked by the quadrotors. **D** Velocity profile, showing peak speeds as a function of time, for the twelve-quadrotor mission of (**B**). Each horizontal row corresponds to a single quadrotor, with color intensity indicating velocity. Stars mark the completion times of individual quadrotors. **E** Trajectory juxtaposing with a decentralized planning method^39^, using eight quadrotors. The mission start and goal positions are aligned. **F** Goal arrival time of each robot.

**Fig. 4:**
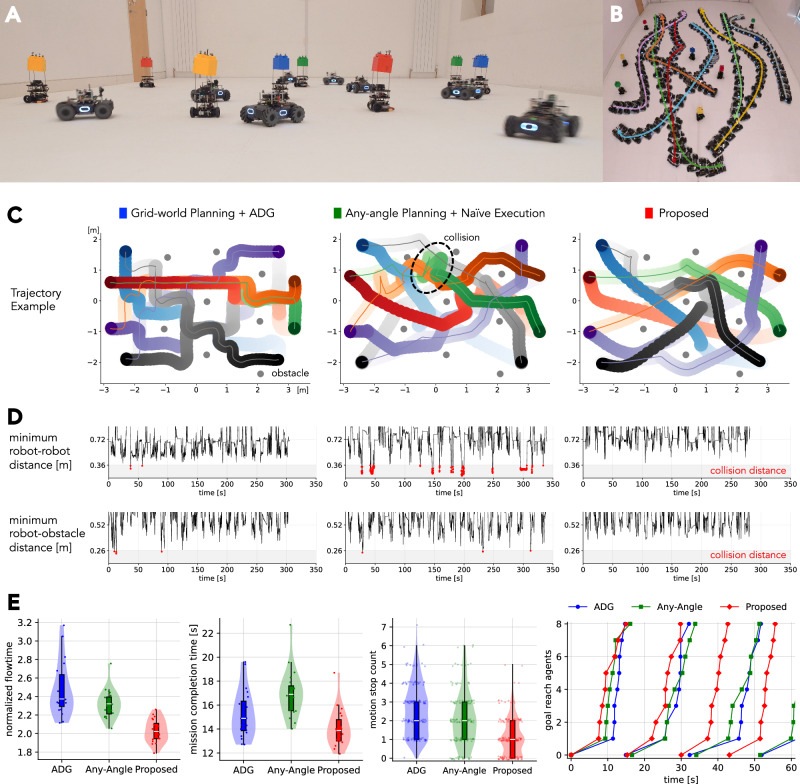
Lifelong mission for ground robots. **A** Experimental setup. Using eight controlled robots and eight moving obstacles, we conducted 20 consecutive, uninterrupted missions for each method. **B** Timelapse trajectory illustration of the proposed method. **C** Qualitative trajectory comparison for the three considered coupled approaches, for a given task configuration (same start and end positions). **D** Safety analysis over the entire mission period, showing minimum distances between two robots and between robots and obstacles. Collisions are marked with red dots. **E** Performance evaluation. The figure shows the normalized flowtime, mission completion time, stop frequency, and the number of targets reached within one mission along the time axis. The flowtime indicates the navigation completion time per robot.

**Fig. 5:**
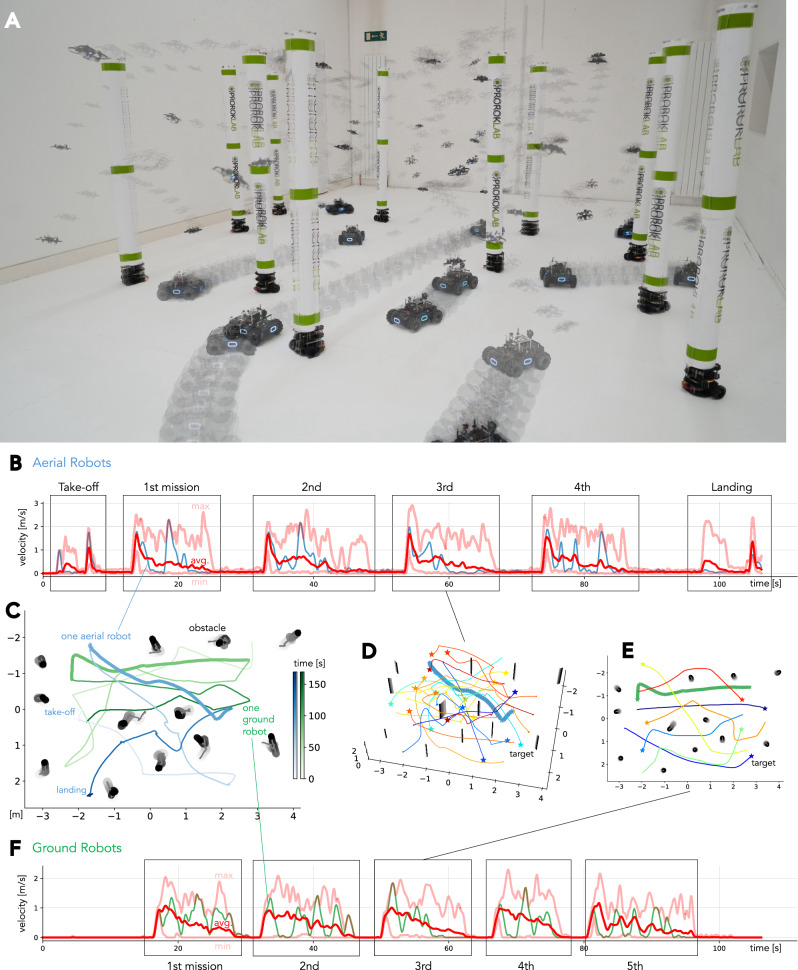
Deployment of 40 robots. **A** Snapshot of the large-scale robot deployment in the compact space, including 20 quadrotors, 8 ground robots, and 12 moving obstacles. **B** Velocity profile for quadrotors. Average, minimum, and maximum speeds are shown with red lines. One quadrotor is highlighted in blue. **C** Top-down trajectory of a single robot for each platform over the entire deployment period. **D** Trajectories for 20 quadrotors within one mission. **E** Trajectories for 8 ground robots. **F** Velocity profile for ground robots. The quadrotors and ground robots move asynchronously in mutually exclusive workspaces.

**Fig. 6:**
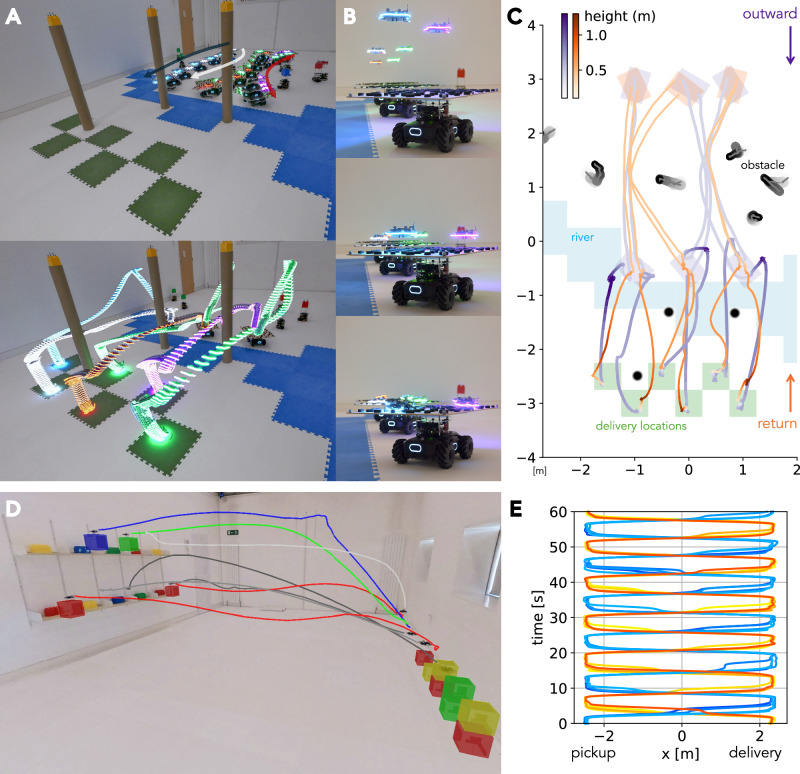
Last-mile delivery and warehouse automation applications. **A** Outward journey of the last-mile delivery scenario. Three ground robots, each carrying two quadrotors, navigate to a river bank, avoiding several moving obstacles. Once arrived, the quadrotors take off from the ground robots and deliver parcels to their respective green-colored delivery points. **B** Quadrotor landing snapshots. Randomly placed, sturdy pads prevent the quadrotors from slipping off the ground robots. **C** Top-down view of the workspace illustrating the entire delivery sequence. Once the delivery is complete, the quadrotors return to their respective ground robots before the entire fleet returns to its starting position. The workspace contains dynamic obstacles that preclude pre-mission planning. **D** Snapshot of the warehouse automation scenario. Quadrotor trajectories and objects carried are overlaid. **E** Eight quadrotor trajectories along with time for one minute, visualizing task progress.

Building upon this theoretical foundation, we develop a multi-robot motion planning and control framework that facilitates reliable multi-robot navigation with a large team of robots. In particular, in known environments with static or slowly changing obstacles, this framework is designed to solve one-shot missions with a minimal pre-planning stage that lasts just a few seconds, while still allowing aggressive motion execution. The simplicity of our solution enables its deployment in a variety of settings involving a variety of robotic platforms and task scenarios (Movie 1). Indeed, using the robotic equipment shown in Fig. 2A, B, we demonstrate (i) aggressive team flights with quadrotors (Fig. 3), (ii) lifelong operation of ground robots with minimal inter-mission delays (Fig. 4), (iii) deployment of 40 robots including both aerial, ground, and moving obstacle robots (Fig. 5), (iv) heterogeneous robot collaboration in a last-mile delivery scenario, and (v) a warehouse automation scenario using aerial robots (Fig. 6).

Despite the confined space and the necessity for tight coordination in these scenarios, we demonstrate the robot teams’ ability to execute agile maneuvers. For instance, the quadrotors achieve a maximum speed of 5.3 m/s, even with 12 airborne robots whose trajectories are intentionally designed to interact. This showcases aggressive quadrotor coordination, significantly exceeding state-of-the-art navigation speeds (which are typically under 2.0 m/s for indoor team navigation, also as shown in Figure S1 in the Supplementary Materials). Furthermore, the 40-robot demonstration, while presenting a significant challenge in terms of scale, density, and engineering complexity, operates continuously beyond a single run, proving the framework’s reliability. Collectively, these demonstrations provide compelling evidence of the practical capabilities of concrete planning.

Designed for real multi-robot fleets, our framework uses *concrete* planning as its core, integrating essential engineering considerations for large-scale operations in complex environments. It specifically tackles real-world challenges frequently neglected in studies that primarily use numerical simulations or small-scale hardware experiments. This focus leads to the architecture presented in Fig. 2C. At a high-level, our framework comprises (i) a multi-agent path planner, which forms our theoretical basis, (ii) a decoupled instantaneous reference trajectory generator, and (iii) a reference tracking controller incorporating reactive collision avoidance as a final safety measure. The latter two components operate within continuous state spaces to generate smooth motion, whereas the planner uses the concrete state representation to achieve rapid, guaranteed coordination at scale. Information flowing downstream, such as the spatiotemporal predictions utilized in concrete planning, is integrated into upstream processes through learnable components trained on real robot trajectory data.

More specifically, given the current configuration and the robots’ destinations, the coordinated path planner (i) generates collision-free and deadlock-free waypoint sequences based on the concrete representation. The planner incorporates low-level controller dynamics through a learned uncertainty module trained on platform-specific trajectory datasets. This model predicts spatial deviations and arrival time, resulting in planning that accurately reflects the navigational capabilities and limitations of each platform. Emerging scalable MAPF techniques (e.g., LaCAM) facilitate the timely derivation of moderately scalable solutions, even given the intricate state space construction required for discrete search. Additionally, since the planner functions within a geometric state space and is independent of high-fidelity dynamics, it can synthesize coordination across diverse robot platforms, thereby cultivating significant architectural transferability.

The reference trajectory generator (ii) then transforms geometric waypoint sequences into dense state trajectories that are kinodynamically feasible and directly accessible to the lower-level controller. In addition to incorporating principled constraints, such as actuation limits and safety requirements, we leverage gray-box learning models^35^ to enhance the robot’s dynamics representation through residual learning. The adoption of a transformer-based^36^ model, which mimics time-optimal trajectories derived from numerical optimization, not only reduces the computational burden but also enables onboard trajectory generation in under a millisecond. This efficiency is key, since it allows for the practical neglect of inter-robot time asynchronism when decomposing global joint plans into individual robot trajectories.

Following reference trajectory generation, a model predictive controller (MPC) (iii) tracks the reference trajectory while minimizing deviations and control effort. The MPC uses nonlinear dynamics models for precise tracking and to enable aggressive robot maneuvers. To ensure safety during deployment, Control Barrier Functions (CBFs)^37,38^ are integrated as hard constraints to promote collision-free motion. CBFs offer an additional safety layer by using local observations to handle unforeseen events, such as dynamic obstacles or model inaccuracies.

This *hierarchical* framework for large-scale multi-robot coordination realizes the potential of concrete planning despite inherent real-world constraints such as imperfect communication, localization uncertainty, and limited computational resources. The architecture adopts a *hybrid* approach that not only bridges the gap between continuous and discrete planning representations, but also integrates first-principles-based methods with data-driven techniques, and centralized planning with distributed (possibly decentralized) execution. By harmonizing these opposing paradigms, our framework overcomes the limitations inherent in each extreme approach. The *modular* design—comprising three purpose-built, decoupled components—is particularly important from an engineering perspective, enabling the construction of large and complex systems, such as the unprecedented 40-robot demonstration reported in this study. While not designed for highly time-varying environments with fast-moving dynamic obstacles, as the following experiments will reveal, such *hierarchical, hybrid, and modular* multi-robot planning schemes offer a compelling solution for realistic operating conditions, potentially establishing them as the new de facto standard in large-scale robotic coordination.

## Results

The framework was deployed on a robot fleet comprising both aerial and ground robots presented in Fig. 2A, to validate its multi-robot motion planning and control capabilities. All experiments were conducted in a compact indoor workspace of 7.0 × 5.0 × 1.8 m^3^, as illustrated in Fig. 2B. The control flow is illustrated in Fig. 2C, which is subsequently described in detail in the Methods section. The detailed hardware specifications are available in Table S1 in the Supplementary Materials.

The primary experiments comprise five scenarios: (i) aggressive quadrotor swarm flights in confined spaces, to verify that concrete planning accommodates continuous dynamics; (ii) lifelong ground robot navigation in gradually changing environments, to verify mission-liveness, where the robots need to navigate between random start-goal locations over extended periods; (iii) large-scale deployment of 40 robots, including both aerial, ground, and obstacle robots, to evaluate system scalability and architecture robustness; (iv) a last-mile delivery scenario using heterogeneous robot teams, to demonstrate collaborative capabilities, and; (v) a warehouse automation mockup scenario with agile quadrotors, to further validate the applicability of our framework to real-world use-cases. We then perform ablation studies to justify the design choices in our framework. These comprehensive results demonstrate the framework’s capabilities, underpinned by the concrete planning, to solve tight and coupled multi-robot coordination under real-world conditions.

### Aggressive flight with a quadrotor swarm

The initial experiment in Fig. 3 (Movie S1) entails a confined space with static obstacles, wherein multiple quadrotors are partitioned into subgroups and situated on the sides of the workspace. The mission objective is for each subgroup to traverse the workspace and reach the opposite side with minimal travel time. The MAPF planning period was set to 2 seconds.

The workspaces are designed with the deliberate intention of compelling the quadrotors to encounter one another at the center of the room. Without coordination, high-density regions emerge that can lead to high-speed collisions and subsequent catastrophic failure. Achieving aggressive maneuvers requires intelligent spatiotemporal region management that accounts for motion uncertainty and quadrotor kinodynamics such as thrust, attitude limitations, and smooth transitions between ascents and descents.

Our experimental validation employs teams of eight and twelve quadrotors (Fig. 3A, B), illustrating the sophisticated, coordinated navigation of all robots to designated goals. Figure 3C presents the system illustration that integrates three components into the deployment framework, using the eight-quadrotor case. The velocity analysis in Fig. 3D highlights the aggressive motion that the quadrotors achieve, even in this packed situation, with peak velocities exceeding 5.0 m/s. This surpasses existing indoor team flights for multi-robot navigation tasks, which typically operate at speeds under 2.0 m/s, with smaller team sizes of less than eight, and in sparser workspaces; Fig. S1 in the Supplementary Materials contextualizes this standout performance among more than 30 state-of-the-art works.

Figure 3E and Movie S6 further highlight the coordination aggressiveness achieved by juxtaposing the robot maneuvers resulting from our framework with those resulting from an established decoupled quadrotor planner^39^. The adoption of coupled planning that elicits robot agility is able to substantially reduce mission completion time, as shown in Fig. 3F. This maneuvering aggressiveness is due to the capability of our framework to provide tight coordination solutions, which are not found by the alternate decoupled planning strategy (providing empirical evidence for the schema presented in Fig. 1). The details and further analysis are provided in Section I of the Supplementary Materials.

### Lifelong operation in gradually changing workspaces

Next, we evaluate the framework’s mission liveliness capabilities in a task for which ground robots must perform persistent navigation between randomized start-goal locations. The experimental setup consists of 20 consecutive uninterrupted missions among eight dynamic obstacles that move randomly within the workspace, as shown in Fig. 4A (Movie S2). Each mission requires four robots to move from one side to the other and vice versa, forcing situations that require non-trivial coordination of collision and deadlock avoidance.

The objective is to maximize the number of completed navigation tasks within a fixed time window. As in the previous scenario, consideration of accurate kinodynamics and uncertainty is mandatory to speedily and safely complete successive missions. Moreover, the system requires planning operations to be completed within a few seconds. This real-time capability is crucial for ensuring continuous operation and maximizing navigation efficiency, and also for adapting to dynamic environments where static planning configurations can quickly become outdated. The existence of dynamic obstacles adds another layer of complexity, demanding adaptive behavior during plan execution—a factor typically not accounted for in the initial coordinated plan generation.

We evaluate our method alongside two commonly used coupled approaches: (i) grid-world MAPF augmented with deadlock-free execution using Action Dependency Graphs (ADG)^40–42^, and (ii) direct execution of any-angle and continuous-time multi-robot path planning^43–45^. For these methods’ plan execution, the robot speed is adjusted to be competitive with ours using trapezoidal velocity profiles. Each high-level planner was given 3 seconds to optimize navigation plans. Visual comparison of resultant robot trajectories is depicted in Fig. 4B, 4C, which highlight the motion smoothness achieved through our method. This is a consequence of the kinodynamic awareness of the high-level path planner. Further descriptions and discussions of these methods are available in Section H of the Supplementary Materials.

Quantitatively, the performance evaluation is conducted in terms of both safety and navigation efficiency. The safety analysis in Fig. 4D examines minimum inter-robot distances and robot-obstacle clearances throughout the whole mission duration. Our proposed framework maintains safe navigation over extended periods, while both baseline methods experience multiple collision events. This is due to their inability to account for kinodynamics in the high-level planner, and also the lack of low-level controller adaptivity in dynamic environments. Figure 4E highlights the navigation efficacy of our framework in terms of three metrics: normalized flowtime, which gauges the average travel time within a single mission; motion stop count, which denotes the number of times the robot pauses during a mission; and mission completion time for all robots. The figure further provides a detailed breakdown of the first 60 seconds, indicating that our framework guides more robots to their destinations in a shorter travel time. As a result, the framework completes 20 consecutive navigation tasks in 270 seconds, outperforming baselines of 310 seconds and 340 seconds. Taken together, these results demonstrate that our framework can complete navigation missions in a faster and safer manner.

### Deployment of 40 robots in a compact space

Using a heterogeneous swarm comprising 40 robots in total (8 ground robots, 20 quadrotors, and 12 dynamic obstacles), we task our method to complete coordinated motion planning in a highly confined space. The ground robots and quadrotors are initially positioned in two groups at opposite ends of the room, similar to the previous scenario, while the moving obstacles were distributed throughout the room (Fig. 5A; Movie S3). The objective is to continuously navigate these robot fleets to assigned targets, which are randomly selected from the opposite room ends. While quadrotors and ground robots do not share the same operational space, they do share the same dynamic obstacles as they span the full height of the experimental space.

On top of the numerous challenges involved in the previous scenarios, this densely populated scenario introduces a considerable degree of intricacy that is not immediately apparent. (i) The presence of dynamic obstacles precludes any offline preparation, thereby necessitating the system’s ability to manage large teams of both ground and aerial robots on demand in response to real-time feedback; (ii) severely limited space due to the high robot count forces continuous, tight coordination; (iii) the entire implementation, encompassing both software and hardware, must exhibit exceptional reliability to facilitate consecutive multi-robot navigation requests at this scale. This imperative arises because, given the nature of mobile multi-robot systems, localized navigation failures can potentially propagate into catastrophic fleet-wide disruptions. Specifically, preventing network traffic spikes, which risk temporarily isolating robots and thereby precipitating system-wide failures, is paramount. Collectively, this scenario subjects multi-robot navigation systems to extraordinary pressure from all directions, establishing it as a definitive testbed for our framework and, by extension, for concrete planning.

The experimental results, illustrated in Fig. 5, demonstrate successful navigation tasks for all 28 actively controlled robots without requiring architectural modifications. All system modules maintained their functionality at scale, executing navigation tasks safely and efficiently without any collisions or deadlocks over repeated trials. Figure 5B, 5F provide velocity profiles of aerial and ground robots, including four and five consecutive runs, respectively. We note a slight reduction in maximum velocity, compared to less cluttered environments, as we employed conservative parameter configurations. Still, the quadrotors achieve a velocity of approximately 3.0 m/s, while the ground robots exceeded 2.0 m/s, which remains competitive and even surpasses reported results from the literature (see Figure S1). Figure 5C illustrates the scenario complexity with the non-trivial trajectories of a single robot from each platform during all missions, together with the in-mission trajectories shown in Figure 5D, 5E. In conclusion, our framework effectively addresses this challenging scenario.

### Application scenarios

Two case studies (last-mile delivery and warehouse automation) are conducted to illustrate the framework’s applicability and transferability to different tasks.

#### Last-mile delivery

Our last-mile delivery scenario^46,47^ requires heterogeneous multi-robot collaboration between aerial and ground robots. We exploit the complementary capabilities of the two types of robots—the quadrotors offer rapid navigation capabilities in 3D space but with limited battery capacity, while the ground robots offer endurance and higher payload capacity, albeit with terrain-specific mobility constraints. The experimental scenario is as follows (Movie S4): (i) A fleet of ground robots, functioning as mobile landing platforms, traverses toward the target area, while avoiding dynamic obstacles. (ii) Upon reaching a ‘river bank’, they serve as deployment bases for the quadrotors, which then navigate across the ‘waterway’ to their designated delivery locations (green tiles in Figure 6A). (iii) Following successful delivery, the quadrotors execute a return flight to their respective ground platforms and land (Figure 6B). (iv) The mission concludes with the ground robots returning to their initial positions.

The entire mission trajectories in Figure 6C show that our framework successfully generalizes to this heterogeneous multi-robot collaboration task without requiring any architectural modifications. Note that the addition of docking platforms doubles the footprint of the ground robots compared to previous experiments, requiring the framework to adapt to this new setup. As will be discussed later, the customization of our framework to this setup was minimal, illustrating a notable degree of applicability to configurations not originally intended. This is a consequence of the framework’s modularity and the planner’s high transferability.

#### Warehouse automation

Warehouse automation^1^ is, to-date, one of the most attractive applications for multi-robot motion planning and control, where multiple robots work in parallel and without interruption to deliver packages from origin locations to pre-defined destinations. Such situations often aim to maximize task throughput, i.e., the number of tasks completed per unit of time, which makes agile motion synthesis a highly sought-after capability. In addition, these systems often impose strict safety criteria, which usually require a traceable and verifiable control stack. Currently, these conditions have dictated the deployment of centralized planners. Our framework is capable of satisfying these conditions. Here we provide a mockup assuming warehouse operations in a 3D space (Movie S5).

Figure 6 D shows a snapshot of a group of four quadrotors delivering virtual parcels, with 3D ‘parcels’ overlaid in the visualization, from a shelf on the left to delivery points on the right. The figure also shows another team of four quadrotors that is simultaneously finishing the current delivery task and moving to the shelf for the next batch. Figure 6E visualizes this repeated process over time. Our framework naturally embodies this industry-inspired scenario. Minimal planning time and the resultant aggressive and dense coordination collectively improve the system efficiency, while safety and liveness properties are sustained by long-horizon, guaranteed concrete planning.

### Ablation study and sensitivity analysis

Thus far, the experiments show that the framework based on concrete planning can navigate multiple robots safely yet aggressively in various scenarios. This section conducts an ablation study and sensitivity analysis on the main modules of our framework to evaluate the architectural design. We also intentionally introduce failure scenarios in real-world conditions to test resilience. Specifically, we examine the framework’s behavior when the reactive collision avoidance scheme, CBF, is inactive. We also evaluate the impact on the high-level planner’s uncertainty evaluation modules, which are controlled by $\sigma \in {{\mathbb{R}}}_{\ge 0}$, a quantity representing the confidence in predictions of spatiotemporal deviations. In addition, the analysis examines the planner’s ability in terms of its scalability and ‘anytime-planning’ property, whereby the quality of the planning result improves over time. More details on each role are provided in the Methods section.

#### Role of reactive collision avoidance

**Fig. 7:**
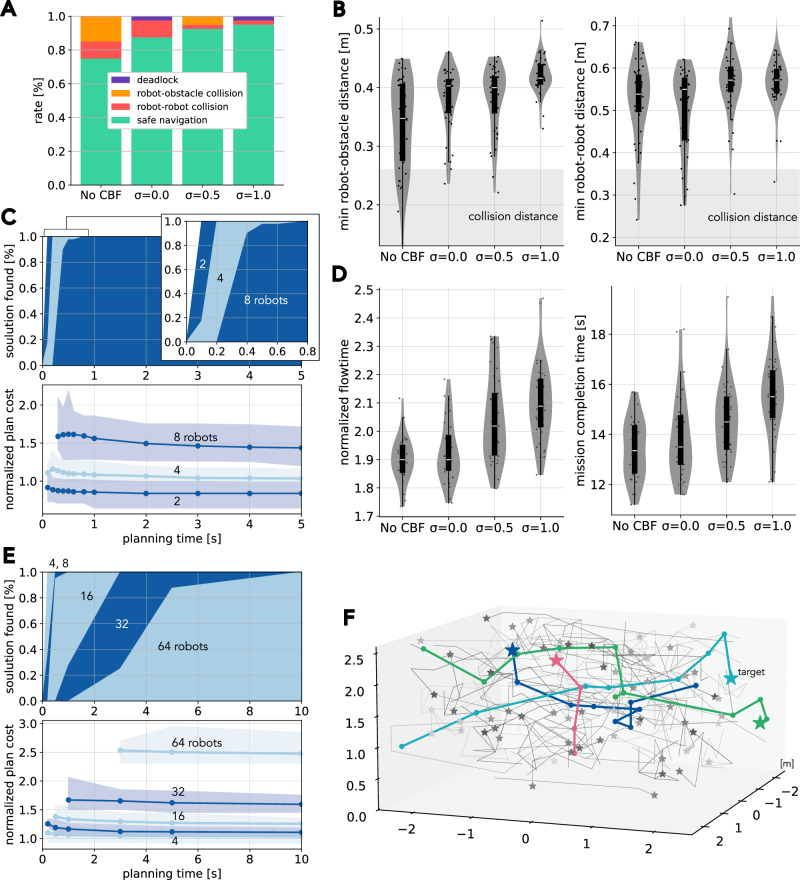
Ablation study and sensitivity analysis. **A** Breakdown of 40 missions. These missions consisted of 8 real ground robots and 8 moving obstacles, identical in configuration to Fig. 3A. **B** Safety measurements regarding inter-robot and robot-robot distances, subject to different uncertainty levels *σ*. **C** Planner success rate and its outcome quality over time using different numbers of ground robots. 40 instances were taken from real robot experiments and tested for each. The planning costs correspond to the flowtime metric. The figure consists of average, minimum, and maximum values. **D** Flowtime and mission completion time with actual runs. **E** Planner scalability assessment in 5 × 5 × 2.5 m^3^ empty space for quadrotors. Each setting was evaluated with 40 randomly generated instances. The planner, with identical parameter configurations to Fig. 5, successfully found solutions for all instances within 10 seconds. (**F**) Generated MAPF plans for 64 quadrotors. Several quadrotors are colored for visibility.

The framework utilizes CBF as the local reactive collision avoidance module due to its reliability and ease of integration into the MPC controller^38^. Through 40 randomized trials with 8 real ground and 8 obstacle robots, identical configuration to Figure 4, Fig. 7A shows that CBF greatly reduces the chance of collision, especially robot-obstacle collisions. In fact, the collision rate exceeds 20% when CBF is removed from the MPC controller. This is reasoned by the reduction in inter-robot and robot-obstacle distances in comparison to their counterparts with CBF, as illustrated in Figure 7B. Conversely, we observe that the removal of CBF can enhance navigation efficacy, as evidenced by the flowtime and mission completion time reduction in Figure 7D. These findings indicate a trade-off between motion aggressiveness and safe navigation. Our framework design emphasizes safety by including CBF to shield robots against unforeseen events such as the nearby presence of dynamic obstacles.

It should be noted that while CBF provides a theoretical guarantee of safety, it exhibits limitations in practical implementations, which may result in collision events under certain circumstances. This happens because the scenario includes increased uncertainties with 8 dynamic obstacles, which can turn feasible plans at the start of planning into infeasible ones during mission execution. For further discussion on this, see Section M in the Supplementary Materials.

#### Role of uncertainty evaluation in high-level planning

The uncertainty module encourages the planner to follow an uncertainty-aware coordination plan, which allows for more conservativeness when robot actions entail large deviations in either space or time. The uncertainty is represented by a Gaussian distribution, thereby enabling the planner’s degree of conservatism, which is controlled by adjusting the distribution level, denoted with *σ*. Figures 7A-D present sensitivity analyses on this parameter *σ*. We observe that higher uncertainty values result in safer navigation styles while having an adverse effect on the aggressiveness of the robot motion. This phenomenon is inductive from the prior debate surrounding the role of CBF. Meanwhile, the excessive conservatism inherent in the motion plan may also result in failure cases, given that the tested scenario encompasses the dynamic obstacles that could potentially cause deadlock scenarios. Indeed, Figure 7A with *σ* = 1.0 includes such a deadlock case. The parameter *σ* was thus set to 0.5 in our reported experiments to strike a balance between the two perspectives.

#### Planner and optimization performance

Figure 7C evaluates the planner’s real-time performance, in terms of (i) the ability to achieve feasible solutions within a limited amount of time, and (ii) the ability to refine solutions through the utilization of additional time. The plan quality is measured in estimated travel time cost. The planner was able to compute a solution for all tested scenarios, involving two to eight robots, within one second, and then continue to refine these solutions. However, the returns on this investment diminish over time, and thus the preceding experiments adopt the early retrieval of solutions from the anytime planning process.

#### Scalability tests

While our framework has already demonstrated direct control of 28 robots with 12 moving obstacles in the real world, we continue to stress test its scalability to higher numbers of robots. Since the individual robot controller, including the reference trajectory generator, is completely decoupled, the only scalability bottleneck lies with the coupled planner. Therefore, we evaluate the scalability of the planner using simulated quadrotors, up to 64, in a compact space with dimensions of 5.0 × 5.0 × 2.5 m^3^. Figure 7E and F show that the planner is capable of handling several tens of quadrotors with a reasonable planning time, i.e., in under 3 seconds, despite its awareness of kinodynamics and uncertainty in the robots’ motions. This scalability is a direct consequence of the adaptation of the advanced MAPF algorithm that we use in our framework, which is capable of handling thousands of agents in real-time in simplified grid-worlds. Section G of the Supplementary Materials further provides empirical data that the planner can address several hundred robots in minutes.

#### Attempts with alternative navigation schemes

We also tested a variant of our framework *without* a coupled MAPF planner. Through 20 randomized trials in an identical setting to Fig. 7A, this ablated version encountered deadlocks in all trials, rendering mission completion impossible. An end-to-end learning approach was also attempted, using a prevalent multi-agent proximal policy optimization (MAPPO) algorithm with graph neural network (GNN-based) communication^48^. The results show frequent collision events, primarily due to the sim-to-real gap and the generalization challenge to unanticipated circumstances. These pilot studies strongly motivate the incorporation of a coupled component (such as ours) to provide coordination guarantees, particularly in situations where sophisticated and reliable coordination is mandatory, as showcased by our series of experimental environments.

## Discussion

We presented the capabilities of *concrete* multi-agent path planning, a holistic framework that uses a discrete representation for combinatorial search while explicitly incorporating continuous robot dynamics. Our deployment framework for point-to-point multi-robot navigation centers on *guaranteed* planning, using modular components seamlessly connected by data-driven artifacts. Although there is a substantial body of work in this domain, our study distinguishes itself through its experiments with 40 robots simultaneously, including both 20 aerial and 8 ground robots, operating at high speeds, and 12 moving obstacles, in a compact space, beyond one-shot tasks, in time-varying environments that preclude offline one-shot planning and dictate the need for mission-liveness. The experiments indicate that *hybrid* multi-robot planning schemes, such as the one proposed here, offer a compelling solution for industrial operating conditions, potentially establishing them as the new de facto standard in large-scale robotic coordination.

Despite challenging practical conditions that often hinder direct transfer of solutions to industrial contexts, we believe that our concrete planning framework is key to facilitating the development of dependable and high-performance multi-robot systems, due to its coordination guarantees and platform-agnostic nature. The application scope encompasses a wide range of domains where real-time coordination of mobile entities is required, including, but not limited to, warehouse automation beyond guided vehicles, logistics operations, and last-mile delivery. Moreover, although not implemented in this study, the quick, coupled planner presented herein opens up the possibility to conduct periodic re-planning, as extensively discussed in the MAPF literature^2,22,25,49,50^, thereby enhancing its capacity to adapt to even more dynamic and uncertain environments.

The concrete representation, beyond mere discrete versus continuous planning, is our foundation, but our successful deployments also stem from the implementation of a hybrid architecture that implicitly integrates diverse navigation paradigms. For example, a high-level coordination sketch is planned in geometric space in a coupled manner, while executable full-state trajectories are obtained in a fully decoupled manner, without consideration of further inter-agent interactions. This intentional shift in planning and representation styles successfully prevents a rapid increase in trajectory planning load, resulting in scalability and mission-liveness.

Data-driven paradigms also play a pivotal role in improving first-principles-based methods by capturing the intricacies of the real-world deployments. For example, reference trajectory generation with imitation learning is introduced to address the need to obtain trajectories immediately after the distribution of a global plan, with no noticeable delay between robots. Given that even with state-of-the-art methods^51–53^ using extensive knowledge of well-studied system dynamics, time-optimal trajectory optimization takes a non-negligible amount of time, the ability to perform sub-millisecond inference is a game-changer from an engineering perspective. Learning from robot trajectories for a better dynamics representation also enhances reliability. Empirical data is available in Section F of the Supplementary Materials.

One of the key challenges of our approach lies in its difficulty of handling highly dynamic environments. While the framework has *low sensitivity* to environmental changes, there is no guarantee that dynamic obstacles that are not considered during the planning phase will be safely avoided at all times (Figure 7A illustrates cases with navigation failures). A moderate number of unforeseen dynamic obstacles will alter the available free space during mission execution, leading to non-negligible discrepancies between the planned and actual robot behavior, hence the occurrence of collisions, particularly when robots are operating at high speeds. Therefore, we argue that the framework is primarily intended for static environments, and the ability to handle dynamic obstacles is designed as a last-resort safeguard. Additional discussion can be found in Section M of the Supplementary Materials. A straightforward workaround to prevent failures in dynamic environments is to adopt conservative CBF parameters that more strictly prevent collisions, coupled with a global monitoring system. If a significant deviation from the planned trajectories is detected, the global planner can be re-invoked. However, dynamically adapting the global plan during aggressive mission executions remains a challenging problem. Not limited to the above, the current framework does not account for completely unknown environments, downwash effects for close proximity flights^54–56^, or non-collaborative and adversarial teams of robots.

This study focuses on advancements in planning and control, and therefore marginalizes the localization problem, deferring it to a remote sensing solution (i.e., an external telemetry system). In principle, our planning and control schemes remain functional as long as they have access to low-latency localization, whether through outdoor GPS or vision-based indoor techniques^57^. We note that our implementation does require a reliable communication scheme for the external state estimation with the motion capture system, and also for the quadrotor swarm control. The majority of failures encountered during the early development process were indeed attributed to communication unreliability. As a result, it became necessary to optimize the distribution of computing resources; the detailed discussion is available in Section E.12 of the Supplementary Materials. Another reliance on communication is the initial distribution of the globally computed plan to each robot, but this incurs a significantly smaller load on the network infrastructure compared to that for state estimation. In addition, our concrete planning solution has a delay tolerance on when to start executing the plan. The empirical data for these aspects (i.e., network utilization and delay tolerance), are available in Sections K and L of the Supplementary Materials.

The experiments encompassed a multitude of scenarios utilizing two distinct robotic platforms. This demonstrates the transferability of the developed framework, which is not limited to a specific robot platform or mission. Indeed, despite its initial design for ground robots, the framework was successfully adapted to aerial platforms without involving any major customization efforts. The flexibility was further demonstrated by the successful implementation of a last-mile delivery demonstration, including new hardware designs, completed within a period of just three days. These rapid developments are due to the platform-agnostic representation used in the concrete planning and the thoughtful modular design, which enables the framework to quickly adapt to new use cases that were not originally intended.

## Methods

**Fig. 8:**
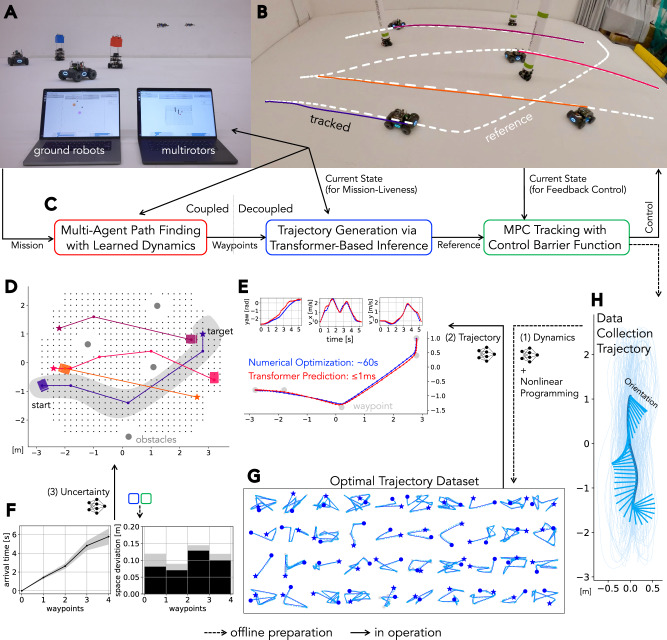
Methodology overview. **A** GUIs for ground robots and quadrotors, allowing users to interactively manage the robot fleet. **B** Overlays for reference and tracked trajectories. **C** Framework pipeline. **D** Discrete waypoints generated by the MAPF planner. The gray-highlighted robot will be used in the next procedure description. **E** Qualitative comparison of the red-colored transformer state trajectory and the blue-colored numerical optimization trajectory. **F** Prediction of arrival time and space deviation used in the high-level planning. The model predicts these values for each waypoint, together with the uncertainty learned from robots' behavior. This information is used for collision checking and cost calculation in the MAPF process. **G** Trajectory dataset preview for training the transformer model. **H** Data collection trajectory for learning residual dynamics, with one segment highlighted. The trained model is used in the optimization-based trajectory generation. In short, we used three machine learning modules. They were trained in the order of the annotations.

Our framework adopts a hierarchical structure (Fig. 8C)—consisting of (i) a multi-agent path planner with learned low-level dynamics, (ii) a learning-based reference trajectory generator, and (iii) a reference tracking controller—that transitions from a high-level discretized planner to distributed controllers in the continuous domain. Its core is the concrete planning, which integrates low-level continuous information into discrete search. This is formulated as multi-agent pathfinding with execution constraints (MAPF-X), enabling robots to coordinate while being agile. Other components of the reference trajectory generator and the MPC controller may be interchangeable, but the framework takes its current form due to practical considerations of reliable deployment of many robots and high transferability to different robot platforms.

This section provides an overview of each module, delineating the technical aspects of deployment, in a bottom-up manner from the low-level to the high-level. This presentation order reflects their interconnected dependencies, unique to our framework, with the high-level component incorporating the low-level feature through data-driven artifacts. In particular, our framework comprises three learning modules: (a) a neural ODE-based residual system identifier, used for training data generation for the reference trajectory generator, that compensates for mismatches between approximated nominal and actual robot dynamics (Fig. 8H); (b) a transformer-based trajectory generator that infers reference states for MPC tracking, designed to imitate optimal trajectories computed by numerical optimization (Fig. 8E, G); and (c) a spatiotemporal motion predictor, used by the MAPF planner, that evaluates the variability in arrival times and path deviations between discrete waypoints (Fig. 8D, F). Section E of the Supplementary Materials provides further technical details, especially for platform-specific designs related to system dynamics.

### Model predictive controller with reactive collision avoidance

The framework employs nonlinear model predictive control (MPC) to compute control commands that follow reference trajectories generated by the upstream process. The inherent robustness to disturbances and reliability in achieving precise control of MPC, grounded in first-principle dynamics $\mathop{\dot{\bf{x}}}={f}_{{\rm{nominal}}}({\bf{x}},{\bf{u}})$, allows the high-level planner to abstract away the complexities of low-level implementation. Here, ${\bf{x}}\in {{\mathbb{R}}}^{n}$ denotes the state vector for a single robot, with ${\bf{u}}\in {{\mathbb{R}}}^{m}$ representing its associated control input.

To prevent inter-agent collisions and facilitate obstacle avoidance, we further integrate a control barrier function (CBF)^37^, denoted by *h*(**x**), into the MPC formulation to define safety constraints. In essence, CBF acts as a fail-safe mechanism, guiding the controller to synthesize safer outputs, relying solely on local observations. This last-resort constraint provides resilience against potential discrepancies in the system models, communication delays, and unexpected events such as time-varying obstacle configurations.

Formally, the control command **u**_0_ is obtained by solving the following numerical optimization with receding horizon at high frequency, which takes a reference state trajectory ${{\bf{x}}}_{{\rm{ref}}}\in {{\mathbb{R}}}^{n\times {N}_{{\rm{MPC}}}}$ and feedback information ${{\bf{x}}}_{{\rm{current}}}\in {{\mathbb{R}}}^{n}$ as input, with hyperparameter $\gamma \in {{\mathbb{R}}}_{\, > \,0}$ and control intervals Δ *t*.

1 $$\begin{array}{lll}\mathop{\min }\limits_{{{\bf{x}}}_{k},{{\bf{u}}}_{k}}\,\mathop{\sum }\limits_{k=0}^{{N}_{{\rm{MPC}}}-1}J({{\bf{x}}}_{k},{{\bf{x}}}_{{\rm{ref}}},{{\bf{u}}}_{k})\\ {\rm{subject}}\,{\rm{to}}:\,{{\bf{x}}}_{k+1}={{\bf{x}}}_{k}+{\int }_{0}^{\bigtriangleup \,t}{f}_{{\rm{nominal}}}({{\bf{x}}}_{k},{{\bf{u}}}_{k})dt,\,{{\bf{x}}}_{0}={{\bf{x}}}_{{\rm{current}}},\\ \mathop{h}\limits^{\circ }({{\bf{x}}}_{k},{{\bf{u}}}_{k})+\gamma h({{\bf{x}}}_{k})\ge 0,\\ {g}_{{\rm{platform}}}({\bf{x}},{\bf{u}})\ge 0\end{array}$$

The integration is performed using the fourth-order Runge-Kutta scheme. The cost function *J*, the mathematical background of CBF, and platform-specific constraints *g*_platform_ appear in the Supplementary Materials.

### Learning-based system identification

MPC requires the reference state trajectory **x**_ref_. In order to design kinodynamically aggressive trajectories that exploit the hardware capability, it is necessary to solve trajectory optimization problems over longer time horizons. This open-loop process requires accurate dynamics models; otherwise, the cumulative errors over the planning horizon will break the trajectory feasibility. This work thus employs a gray-box dynamics representation in trajectory optimization, whereby a portion of the robot’s dynamics is modeled from first-principles and further refined with residual dynamics obtained through data-driven components.

In particular, inspired by data-driven gray-box controller designs^58,59^, the framework uses neural ordinary differential equations (NODE)^35^, which learn residual and continuous dynamics in time so that we can later use them for numerical integration with a tunable discretized time step. Formally, our dynamics modeling is expressed as *f*_gray_(**x**, **u**) = *f*_nominal_(**x**, **u**) + *f*_NODE_(**x**, **u**), where the residual dynamics is learned by a neural network parameterized by *θ*_NODE_. To obtain *f*_NODE_, we first collect trajectory data ${\{({{\bf{x}}}_{k},{{\bf{u}}}_{k},{\bigtriangleup t}_{k})\}}^{k=0,1,\ldots ,M}$, where ${\bigtriangleup t}_{k}\in {\mathbb{R}}$ represents the sampling period, consisting of over 1,000 seconds of robot motion for each platform. The data was collected by tracking specially designed reference trajectories (Fig. 8H) that uniformly cover the robot’s state space using MPC. The training then minimizes the prediction error of the state transitions:

2 $${\mathcal{L}}({\theta }_{\mathrm{NODE}})=\frac{1}{M}\mathop{\sum }\limits_{k=0}^{M-1}{\left\Vert {x}_{k}+{\int }_{0}^{{\bigtriangleup t}_{k}}{f}_{\mathrm{gray}}({{\bf{x}}}_{k},{{\bf{u}}}_{k})dt-{{\bf{x}}}_{k+1}\right\Vert }_{2}$$

Once the model is trained, we can use the high-fidelity dynamics *f*_gray_ in numerical integration.

It should be noted that the learning-based system identification is performed solely to improve reference trajectory generation. We do not use *f*_gray_ in the MPC for two reasons: (i) By incorporating *f*_gray_, the MPC optimization problem becomes significantly more complex, as the resulting dynamics constraints introduce strong nonlinearities and increase the computational burden. Compounded with CBF constraints as hard safety requirements, it leads to longer computation time and could cause unstable behavior. The slow convergence speed and potential sub-optimal solution is incompatible with real-time control with aggressive maneuvers. (ii) Instead, MPC with *f*_nominal_ enables rapid recomputation using real-time state feedback, thereby compensating for model errors. In short, we sacrifice some model accuracy to gain a faster resampling rate in the closed-loop control. Even when *f*_nominal_ is used in the MPC, a high-fidelity reference trajectory allows robots to maneuver aggressively while maintaining moderate tracking errors that do not compromise execution. See Section F of the Supplementary Materials for empirical evidence.

### Reference trajectory via numerical optimization

Given a sequence of geometric waypoints $[{{\rm{p}}}_{1},{{\rm{p}}}_{2},\ldots ]\in {{\mathbb{R}}}^{3\times {N}_{{\rm{waypoint}}}}$ from the upstream process, the framework formulates a generation of kinodynamically aggressive reference state trajectories as a trajectory optimization with a nonlinear formulation to minimize the travel time, akin to an autonomous drone racing scenario^51^. The notable difference, however, is that large spatial deviations from the straight line between two consecutive waypoints are discouraged, as such deviations make tight coordination impossible in dense environments.

In particular, we first fit the waypoint sequence with linear interpolation and sample a ‘denser’ waypoint sequence $[{{\rm{p}}}_{1}^{{\prime} },{{\rm{p}}}_{2}^{{\prime} },\ldots ,]\in {{\mathbb{R}}}^{3\times {N}_{{\rm{traj}}}}$, where *N*_traj_ ≫ *N*_waypoint_. Given initial state **x**_init_, terminal states *X*_fin_, and deviation tolerance ${\overline{d}}_{{\rm{traj}}}\in {{\mathbb{R}}}_{\ge 0}$, the following formulation derives the desired trajectories using *N*_traj_ state and control variables, **x**_*k*_ and **u**_*k*_, associated with the sampling time Δ *t*_*k*_.

3 $$\begin{array}{lll}\mathop{\min}\limits_{{{\bf{x}}}_{k},{{\bf{u}}}_{k},{\bigtriangleup t}_{k}}\,\mathop{\sum}\limits_{k=0}^{{N}_{\mathrm{traj}}-1}{\bigtriangleup t}_{k}\\ \mathrm{subject}\,\mathrm{to}:\,{{\bf{x}}}_{k+1}={{\bf{x}}}_{k}+{\int}_{0}^{{\bigtriangleup t}_{k}}{f}_{\mathrm{gray}}({{\bf{x}}}_{k},{{\bf{u}}}_{k})dt,\,{{\bf{x}}}_{0}={{\bf{x}}}_{\mathrm{init}},\,{{\bf{x}}}_{{N}_{\mathrm{traj}}-1}\in {X}_{\mathrm{fin}}\\ \parallel {{\rm{p}}}_{k}^{{\prime}}-{\rm{p}}({{\bf{x}}}_{k}){\parallel}_{2}\le {\bar{d}}_{\mathrm{traj}}\\ {g}_{\mathrm{platform}}({{\bf{x}}}_{k},{{\bf{u}}}_{k})\ge 0\end{array}$$

Platform-specific constraints *g*_platform_ can encode physical constraints in addition to the gray-box dynamics, leveraging our apriori knowledge based on per-robot deployments, such as actuation limits and safety requirements. This optimization ensures that the generated state trajectory intersects with the waypoints from the high-level planner, thereby maintaining consistency between the path planning and the trajectory generation. The resulting trajectories are then parameterized using spline curve interpolation to match the sampling rate to other processes.

Our implementation involves extensive grid search over hyperparameter sets consisting of the number of variables *N*_traj_ and initial solution guesses. This approach is designed to obtain solutions reliably, independent from specific problem instances, without manual tuning, despite the complex nonlinearity caused by constraints and gray-box dynamics. However, due to its computational overhead, even with a simple instance as shown in Fig. 8C, the amount of time required to derive solutions is unpredictable. This renders the mere numerical optimization incapable of hosting mission-liveness, especially in gradually changing environments. The lack of real-time property leads us to replace the precise trajectory optimization with a constant-time approximation by the learning-based alternative.

### Reference trajectory generation via transformers

The aforementioned problem of heavy computation is addressed by implementing imitation learning inference. This approach is justified by the fact that the precise reference trajectory is not a prerequisite due to the inherent capability of MPC.

In particular, the framework employs transformers^36^ as its backbone architecture, and then translates the dense waypoint sequence $[{{\rm{p}}}_{1}^{{\prime} },{{\rm{p}}}_{2}^{{\prime} },\ldots ,]$ into a corresponding state trajectory $[{{\bf{x}}}_{1}^{{\rm{pred}}},{{\bf{x}}}_{2}^{{\rm{pred}}},\ldots ]$, with time assignment $[{t}_{1}^{{\rm{pred}}},{t}_{2}^{{\rm{pred}}},\ldots ]\in {{\mathbb{R}}}^{{N}_{{\rm{traj}}}}$ to each state. The transformer is capable of processing complex arbitrary-length time-series data^60^, utilizing a self-attention mechanism that considers temporal correlations across all states. These features are of paramount importance for the generation of smooth and dynamically feasible motion profiles, which apply to various robot platforms^61,62^.

Through numerical optimization, we generated a substantial trajectory corpus, comprising approximately 100,000 instances for each robot platform, using over 300 CPU cores with extensive parallelization. The dataset preview is shown in Fig. 8G. Million-parameter models were trained using the ordinary mean square error as imitation loss. This model size can capture complex state trajectory shapes in higher dimensional spaces, while still allowing for sub-milliseconds inference on onboard computing devices. Rapid trajectory generation also underpins our scalable quadrotor setups, which can handle 20 simultaneous queries with negligible latency on a single powerful computer. The quality of the generated trajectories is sufficient to fulfill the purpose of our framework. As illustrated in Fig. 8E, even for a previously unseen query, the discrepancies between the ground-truth and the generated trajectory are minor and can be compensated with MPC.

### Coordination guaranteed waypoint sequence generation

This module facilitates coordination by assigning collision-free and deadlock-free waypoint sequences to each robot. Our core invention is the ‘concrete’ formulation of multi-agent pathfinding (MAPF), capable of capturing low-level continuous dynamics while still allowing the use of discrete search, and its planner, which uses advanced MAPF algorithms to derive solutions rapidly. They collectively underpin all the demonstrations presented. Technical details of the formulation called *MAPF-X (MAPF with execution constraints)* and the planner called *Tree-LaCAM* are provided in Sections C and D of the Supplementary Materials. Here we briefly present their intuitions.

Given a geometric roadmap and a start-goal pair for each robot, MAPF aims to assign a sequence of spatiotemporal points to each robot, as shown in Fig. 8D. The standard MAPF formulation defines a robot state as a spatiotemporal location, assuming that all robots take actions synchronously and complete their action exactly in unit time. This commonly used formulation has accelerated multi-agent planning research due to its simplicity^18,21^, but it also makes it difficult to reflect continuous robot motion, especially for aggressive maneuvers. We address this problem by developing a kinodynamic and uncertainty-aware state representation, combined with a data-driven motion model that translates discrete information into a continuous spatiotemporal prediction.

In particular, we adopt a discrete per-robot state space as a combination of: (i) the current time, (ii) the travel edge, i.e., a pair of vertices representing the robot’s departure and arrival points; (iii) a fixed-length trajectory history of visited vertices; (iv) a progress index indicating the time remaining to complete the current move; and (v) uncertainty about the completion time of the current move. The travel time of (iv) is determined on-demand by a prediction model trained on real robot data, using the trajectory history of (iii). This allows smooth motion to be captured beyond the unit-time assumption in classical MAPF. Inter-agent collision checking is performed based on the spatiotemporal overlap of their states, with additional consideration of uncertainty predictions provided by the trained model to encourage safe multi-robot navigation. These prediction examples are shown in Fig. 8F. The MAPF-X problem is to assign each robot a collision-free path with this extended state representation.

Building on a scalable algorithm called LaCAM^24,33,34^ for classical MAPF, we develop a tree-search algorithm called Tree-LaCAM to solve MAPF-X. This algorithm performs an exhaustive search over the joint state space of all robots, while dramatically reducing the search effort through lazy successor state generation. Theoretically, we prove that Tree-LaCAM is *complete* and *optimal* for MAPF-X; in finite time, it finds an optimal solution that minimizes the solution cost if solutions exist, otherwise it reports the nonexistence. In practice, Tree-LaCAM acts as an anytime algorithm that quickly finds an initial solution and gradually refines it over time, eventually converging to the optimal solution. Empirically, the implementation is able to address tens of robots in a few seconds, as shown in our demonstrations. Section G of the Supplementary Materials further shows that with a computation time scale of minutes, it can scale to hundreds of robots, while still accounting for kinodynamics and uncertainty.

This state space definition is inspired by advanced single- and multi-agent planning abstractions to adapt to physical robot constraints^43,44,63–66^. Although these abstractions are typically evaluated in simplified simulations, our approach bridges the gap between theoretical frameworks and practical applications by exploiting recent advances in MAPF techniques and learning from the robot trajectories. With this representation, the framework can generate kinodynamically feasible waypoints governed by physical constraints without explicitly modeling platform-specific state spaces.

### Training motion model

The final component is the motion predictor used in the MAPF process. The model predicts the travel time and the spatial deviation when a robot is about to travel from one waypoint **p**_*t*_ to another **p**_*t*+1_, with help of trajectory history [${{\bf{p}}}_{t-{N}_{{\rm{MAPF}}}}$, ${{\bf{p}}}_{t-{N}_{{\rm{MAPF}}}+1}$, …, **p**_*t*−1_], collectively referred to as *P*_*t*_. The spatial deviation is defined as the maximum distance between the robot’s actual position and the edge 〈**p**_*t*_, **p**_*t*+1_〉.

We create a dataset $\left\{\langle {P}_{k},{y}_{k}^{{\rm{space}}},{y}_{k}^{{\rm{time}}}\rangle \right\}$, where the latter two denote the space and time labels, using the following procedure. (i) A finite waypoint sequence ${P}_{k-l:k+l^{\prime} }=[{{\bf{p}}}_{0},{{\bf{p}}}_{1},\ldots ,{{\bf{p}}}_{\alpha }]$ to which *P*_*k*_ belongs is prepared either by random walks within the arena, or by shortest paths on roadmaps constructed with randomly placed virtual obstacles. The second method provides a closer approximation to real deployment setups to reduce distribution shifts. (ii) The robot then tracks ${P}_{k-l:k+l{\prime} }$ with the transformer trajectory generator and MPC, resulting in the recorded timed trajectory $\pi =[\langle {{\bf{x}}}_{0},{t}_{0}\rangle ,\langle {{\bf{x}}}_{1},{t}_{1}\rangle ,\ldots \langle {{\bf{x}}}_{\beta },{t}_{\beta }\rangle ]$. (iii) A sequential alignment optimization between ${P}_{k-l:k+l{\prime} }$ and *π* is solved to assign each waypoint to one of the states based on the positional difference. This yields a sequence of $\langle {{\bf{p}}}_{i},\langle {{\bf{x}}}_{j},{t}_{j}\rangle \rangle$, which can be directly used to construct the dataset. Upon the data collection, a single neural network is trained to predict $\left\langle {y}_{k}^{{\rm{space}}},{y}_{k}^{{\rm{time}}}\right\rangle$. In particular, the model assumes a Gaussian distribution, and therefore the training is conducted using a negative log-likelihood loss function. Our database contains more than 50,000 samples for each platform, collected over four hours.

## Supplementary information

Supplementary Information

Supplementary Movie 1

Supplementary Movie 2

Supplementary Movie 3

Supplementary Movie 4

Supplementary Movie 5

Supplementary Movie 6

Supplementary Movie 7

## Data Availability

All data, materials, and codes needed to evaluate the paper’s conclusions will be publicly available at https://proroklab.github.io/agile-mapf.
