# Supplementary material for: Concrete multi-agent path planning enabling kinodynamically aggressive maneuvers
Source: Npj Robot. 2026 Mar 14;4(1):20. doi: 10.1038/s44182-026-00083-2 (PMC12988861; doi:10.1038/s44182-026-00083-2)
Supplement: Supplementary file 1 — Supplementary Information [file 44182_2026_83_MOESM1_ESM.pdf]

Supplementary Materials for Concrete Multi-Agent Path Planning Enabling Kinodynamically Aggressive Maneuvers

Keisuke Okumura[12†*], Guang Yang[1†], Zhan Gao[1], Heedo Woo[1], Amanda Prorok[1*]

[1]University of Cambridge, UK
[2]National Institute of Advanced Industrial Science and Technology (AIST), Japan

[*]Corresponding author. Email: {ko393, asp45}@cst.cam.ac.uk
[†]These authors contributed equally to this work.

This PDF file includes:

Hardware specifications
Survey on navigational agility of indoor quadrotor swarm
MAPF-X: Formulation of concrete planning
Solving MAPF-X
Materials and methods
Empirical observation of learning-based reference trajectory generation
Scalability assessment for MAPF-X planner
Baseline implementations for the ground robots experiments
Juxtaposing with decentralized drone control
Onboard CPU and memory usage
Communication analysis
Delay tolerance for distributing global plans
Presence of dynamic obstacles
Figures S1 to S9
Tables S1 and S2
Captions for Movies S1 to S6

Other Supplementary Materials for this manuscript:

Movies S1 to S6

A Hardware specifications

Table S1 lists the hardware details of three types of robots used in our experiments. The ground robots are the Cambridge Robomaster [38], while the aerial robots are a newly built platform. The obstacle robots are based on TurtleBot3. Their appearance is shown in Figure 2.

Table S1: **Hardware specifications.**

**Ground robot**	
Platform	DJI RoboMaster S1
Computing unit	NVIDIA Jetson Orin NX 16 GB module
Carrier board	AVERMEDIA D131
Size	340 mm × 250 mm × 170 mm
Size (with landing dock)	610 mm × 410 mm × 180 mm
Mass	3.3 kg
Maximum velocity	4.45 m/s

**Quadrotor**	
Platform	BetaFPV Pavo Pico
Microcontroller	Seeed Studio XIAO ESP32S3
Flight controller	BetaFPV F4 2-3 S 20 A AIO (Betaflight 4.5.1)
Motors	BetaFPV 1102 14000 Kv
Propellers	45 mm Gemfan
Battery	2S 8.4 V 450 mAh LiPo
Size	160 mm × 160 mm × 35 mm
Mass	0.072 kg

**Moving obstacle**	
Platform	TurtleBot3
Computing unit	32-bit ARM Cortex®-M7 with FPU
Size	138 mm × 178 mm × 192 mm
Mass	1 kg
Maximum translational velocity	0.22 m/s
Maximum rotational velocity	2.84 rad/s

B Survey on navigational agility of indoor quadrotor swarm

To support the motion agility of quadrotors established in our empirical results, Figure S1 summarizes the velocity reported from existing work on indoor multi-robot navigation, along with our results. The figure only includes work that actually deploys physical quadrotors, as this paper's focus is on the deployment. We also exclude work that assumes manually designed trajectories or work that only implements collision avoidance features, as they are not capable of solving multi-robot navigation as studied in this paper.

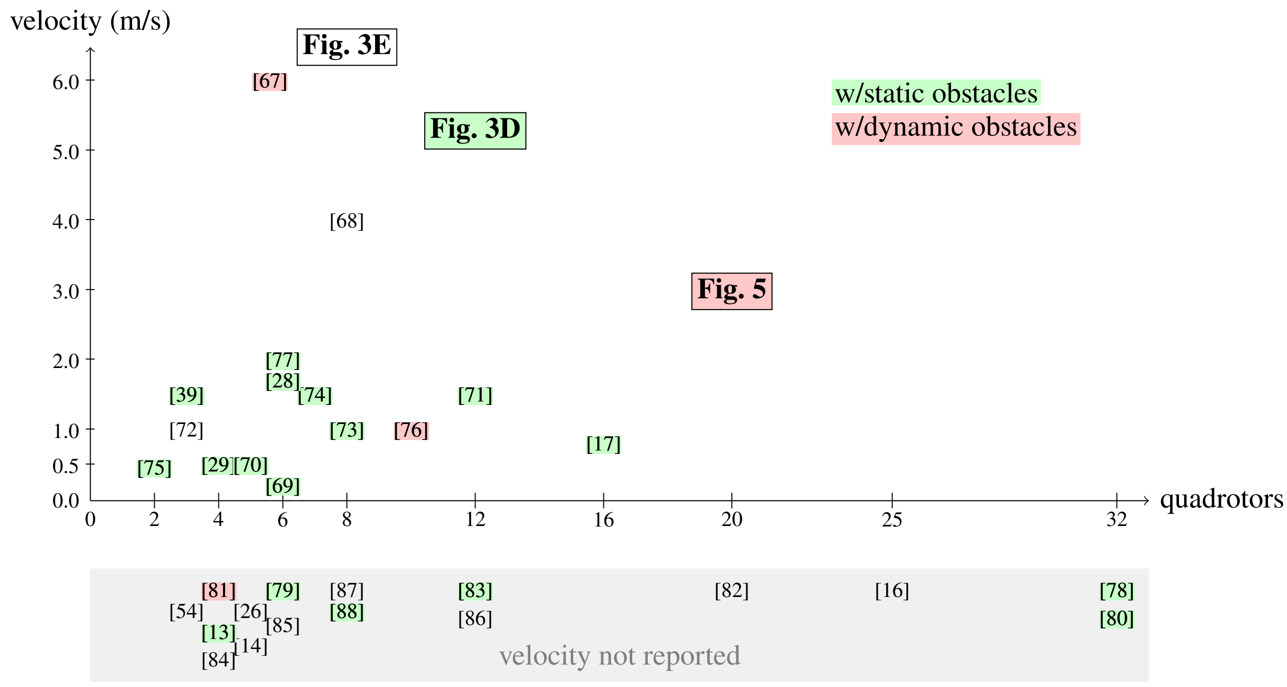

Figure S1: **Navigational agility of indoor quadrotor swarm.** An overview of over 30 references that deploy indoor quadrotors, showing their maximum reported speed, where available, otherwise showing the reported average speed. The references for which we could not find speed information are listed at the bottom.

This survey emphasizes the uniqueness of our study in establishing kinodynamic aggressiveness at this scale, even considering the hardware differences. In particular, the result in Figure 3 outperforms most existing work in terms of speed, although the situations are intentionally designed to involve non-trivial coordination of up to 12 quadrotors. In addition, to the best of our knowledge, the result of Figure 5 represents the largest indoor quadrotor swarm deployment in environments with dynamic obstacles to solve multi-robot navigation tasks, despite its simultaneous deployment of the ground robot fleet. Note that several studies, for which their speed report is not available, have successfully deployed more quadrotors than ours, but we presume that these studies are less aggressive than ours partly due to their platform limitations [89]. Still, the present study is distinguished by its pioneering deployment of large-scale quadrotor teams within dense and dynamic workspaces, while permitting their agile maneuvers.

We have 20 physical quadrotors thus we deployed 20 quadrotors, but at least from the planning and control perspective, our framework is capable of handling more as discussed in the ablation study section. Meanwhile, we modestly claim that the localization and communication perspective may face different problems, as scaling physical systems is not trivial.

C MAPF-X: Formulation of concrete planning

Our exhaustive deployments build on a novel formulation of kinodynamic and uncertainty-aware geometric planning for multiple agents, one of the key inventions of this study. Herein, we call this

problem *MAPF-X*, named after multi-agent pathfinding (MAPF) with execution constratins from physical robot deployments. This section describes the background, formalization, and approaches to solving MAPF-X.

C.1 Classical MAPF and challenges

MAPF [18, 21] is a graph path planning problem for multiple agents that seeks collision-free paths for each agent. Motivated by its industrial applicability, including warehouse automation [1, 2], MAPF has undergone extensive development since the 2010s, and researchers have invented powerful methods, such as planning algorithms that handle hundreds of agents or more in seconds [90, 22, 23, 24]. We leverage these developments to establish a multi-robot motion planning and control scheme with coordination guarantees.

A classical MAPF problem is defined by a geometric roadmap $G = (V, E)$, where V is a finite set of vertices, $E \subseteq V \times V$ is a set of (directed) edges including self-loops, and each agent $i \in \{1, 2, \ldots, n\}$ which is assigned start and goal vertices, $s_i \in V$ and $g_i \in V$, respectively. Each vertex v is associated with a geometric location in $\mathbb{R}^3$. Then, the discrete state space for an agent i is captured as $\langle t, v\rangle$, where $t \in \mathbb{N}_{\geq 0}$ represents the timestep and $v \in V$ the occupying vertex; i.e., agent i is on location v at discrete time t. We denote this state representation Ω_{base}. A solution to MAPF is then agent-wise paths, $\left[\langle 0, v_i^0\rangle, \langle 1, v_i^1\rangle, \ldots, \langle k, v_i^k\rangle\right]$ for agent i, such that (i) $v_i^0 = s_i$, (ii) $v_i^k = g_i$, (iii) $(v_i^t, v_i^{t+1}) \in E$, and (iv) there are no collisions between agents. The definition of collisions varies. The typical formulation defines collisions when two agents are at the same location, or when two agents switch positions within one timestep. For ease of implementation and practicality, most studies assume that G is a four-connected 2D grid.

The above formulation abstracts well the demands from logistics systems, however, its outcome is not ready for 'lively' executing by actual robots living under real-world constraints. More specifically, classical MAPF is sufficiently executable if the target robot systems are single-integrators with no motion uncertainty. Meanwhile, our interest is in establishing aggressive coordination, which forces planners to be aware of kinodynamics and spatiotemporal tracking errors. To do so, we need to extend the state representation of $\langle t \in \mathbb{N}_{\geq 0}, v \in V\rangle \in \Omega_{\text{base}}$.

One possible approach is to use the robot's full state $\mathcal{X} \subset \mathbb{R}^n$, which includes, e.g., velocity, acceleration, orientation, and then conduct the planning over the state representation of $\langle t \in \mathbb{N}_{\geq 0}, x \in \mathcal{X}\rangle$. Indeed, algorithms developed for classical MAPF are easily applied to the full state representation [91, 92]. However, such approaches have problems with both the scalability for the number of agents and the real-time planning capability. This is mainly due to the difficulty of designing an efficient heuristic function beyond geometric state spaces. In addition, such an implementation is often specific to the target robotic systems, reducing engineering transferability.

In summary, we are interested in a state representation for MAPF that is based on geometric space but sufficiently reflects the kinodynamics and motion uncertainties.

C.2 Removing assumptions of unit-time action

To construct MAPF-X, we first need to dismantle the unit-time action assumption that each agent can move from its current location to a neighboring location in exactly one timestep. Such an assumption is feasible only with a grid-world, where every edge has the same length, and with simple robot

dynamics, where robots move at constant speed. The adaptation requires the introduction of edge-dependent travel time into the MAPF formulation, as in [43, 65, 44, 45].

Let us now extend the state space for one agent as $\langle t, e, \tau \rangle$, where:

- $t \in [0, \delta, 2\delta, \ldots]$ denotes the time at the current state. For a smooth connection with the robot deployments, let its unit be seconds. Then, we introduce a small unit of time, $\delta \in \mathbb{R}_{>0}$, which is set to 0.1 seconds in our experiments.
- $e \in E$ represents the edge traversed by the agent.
- $\tau \in [1, 2, \ldots, T(e)]$ is a progress index, representing elapsed time on e. The travel time required for e is determined by $T : E \mapsto \mathbb{N}_{>0}$, whose output is normalized by δ. In other words, $\delta T(e)$ seconds represent the actual travel time.

Given a robot state $\langle t, e, \tau \rangle$, its successor state is $\langle t+\delta, e, \tau+1 \rangle$ if $\tau < T(e)$. If $\tau = T(e)$, this means that the agent finishes the transition along e; therefore its successor state would be $\langle t + \delta, e', 1 \rangle$, where e' is an edge starting at the end of e. Let us denote this state representation Ω_{edge}. When $T(\cdot)$ is always one, Ω_{edge} is identical to Ω_{base}.

Observe that Ω_{edge} incorporates the edge-dependent travel time, still being a discrete representation. Therefore, it is possible to derive a solution path connecting the queried initial and target states using discrete search algorithms such as A*. Similarly, in the multi-agent case, we can apply discrete MAPF algorithms developed for Ω_{base} once we have defined collisions between agents.

Inter-agent collision checking with Ω_{edge} has to be conservative because we do not know exactly where the robot is when it moves along the edge e. Formally, let $\Delta(e) \subset \mathbb{R}^3$ be the footprint region during the robot's traverse at e. Besides, given a state $s \in \Omega_{\text{edge}}$, let $t_{\text{LB}}(s)$ and $t_{\text{UB}}(s)$ be the start and end time of the journey for an edge of s, denoted as $e(s)$ for convenience, which are easily computed from the time information of s. With Ω_{edge}, two states s and s' have collisions when

$$\Big\{ [t_{\text{LB}}(s), t_{\text{UB}}(s)] \cap [t_{\text{LB}}(s'), t_{\text{UB}}(s')] \neq \emptyset \Big\} \quad \wedge \quad \Big\{ \Delta(e(s)) \cap \Delta(e(s')) \neq \emptyset \Big\}, \tag{S1}$$

i.e., two states are considered to collide if they have a spatiotemporal overlap.

C.3 Introducing action history

Ω_{edge} can accommodate edge-dependent travel time, and thus can address the general case of MAPF beyond a grid-world representation. However, the abstraction with Ω_{edge} is not enough to capture aggressive robot movements. This is because the travel time on an edge e actually varies according to the previous actions of this robot due to its dynamics. For example, suppose that a ground robot transits its position as (i) $(0,0) \rightarrow (1,0) \rightarrow (1,1)$, or (ii) $(1,-1) \rightarrow (1,0) \rightarrow (1,1)$ in the Euclidean space. The last action $e : (1,0) \rightarrow (1,1)$ in these two sequences is identical, however, it is natural to assume that the former (i) requires more time for completing e due to its direction change from the previous action. On the flip side, in the latter sequence (ii), the robot completes e faster because it can maintain a high speed at the beginning of e from its previous action. Ω_{edge} that relies on $T(e)$ cannot distinguish between these two situations.

We claim that a more accurate modeling of the travel time needs to take into account the action history, as proposed in [63] for a single agent. Let us make this idea concrete. We extend the state

representation of Ω_{edge} to $\langle t, e = (v_{\text{from}}, v_{\text{to}}), \tau, \pi \rangle$, where the first three are identical to Ω_{edge}, while the last term π represents a fixed-length waypoint history that the agent has recently visited, i.e., $\pi = (v_{-N_{\text{MAPF}}}, v_{-N_{\text{MAPF}}+1}, \ldots, v_{-1}) \in V^{N_{\text{MAPF}}}$. $N_{\text{MAPF}} \in \mathbb{N}_{\geq 0}$ is a hyperparameter. Then, the domain of τ, i.e., travel time for e, is determined by $T : V^{N_{\text{MAPF}}} \times E \mapsto \mathbb{N}$. A successor state inherits π when $\tau < T(\pi, e)$, otherwise, the history is updated to $(v_{N_{\text{MAPF}}-1}, v_{N_{\text{MAPF}}-2}, \ldots, v_{-1}, v_{\text{from}})$. Let Ω_{hist} denote this new state representation. When N_{MAPF} is set to zero, Ω_{hist} is identical to Ω_{edge}.

With Ω_{hist}, it is possible to distinguish between consecutive actions without turning and zigzag actions that require frequent decelerations, since the predicted travel time T also depends on the history of the action. For MAPF, we can also introduce collision checking that reflects robot dynamics better, by adding a dependency of the history π to the footprint mapping Δ, i.e., replacing $\Delta(e)$ in (S1) with $\Delta(e, \pi)$.

C.4 Uncertainty incorporation

Ω_{hist} assumes deterministic functions T and Δ for modeling travel time and footprint, respectively. However, this is not the case for real robot behavior, especially for agile maneuvers, which involve spatiotemporal variations even for the same trajectory tracking. Therefore, both travel time and footprint do indeed follow probabilistic distributions. The introduction of non-deterministic models can also reduce the abstraction mismatch from the continuous robot state in $\mathcal{X}$ to the discrete representation Ω_{hist}.

One consideration, however, is that providing probabilistic safety guarantees for MAPF is computationally very expensive [64, 66, 93] and thus degrades scalable, real-time planning capability. Instead, we aim to construct a deterministic problem formulation that still allows for the incorporation of uncertainty. This is possible with the development of a yes-or-no deterministic collision check function by refining (S1).

With a natural choice, we use Gaussian distribution for modeling footprint and time prediction, denoted as $\mathcal{N}_{\text{space}}(\mu_{\text{space}}, \sigma^2_{\text{space}})$ and $\mathcal{N}_{\text{time}}(\mu_{\text{time}}, \sigma^2_{\text{time}})$. Their parameters, $\{\mu, \sigma\}_{\{\text{space},\text{time}\}}$, are determined by the traveling edge e and the action history π. Note that the following discussion can easily be applied to other distribution choices, such as the beta distribution, and thus the problem formulation remains general.

Let's first consider how to address uncertainty in space regarding the footprint Δ. Given a target edge $e = (v_{\text{from}}, v_{\text{to}})$ and action history π, suppose that $\mathcal{N}_{\text{space}}$ represents a spatial maximum deviation of a line segment between v_{from} and v_{to} in the Euclidean geometric space. The maximum deviation from the line segment is thus expressed as

$$r(e, \pi) = \mu_{\text{space}}(e, \pi) + \alpha_{\text{space}} \cdot \sigma_{\text{space}}(e, \pi), \tag{S2}$$

where $\alpha_{\text{space}} \in \mathbb{R}_{\geq 0}$ is an adjustable parameter to control the conservatism of the collision check. Then the footprint Δ is framed as a capsule-shaped space surrounding e with a diameter of $r(e, \pi)$, i.e.,

$$\Delta(e, \pi) = \left\{ x \in \mathbb{R}^3 \mid ||x - e|| \leq r(e, \pi) \right\} \tag{S3}$$

We now consider the time uncertainty, with the modeling $\mathcal{N}_{\text{time}}$ that represents the edge travel time over e. Unlike space, the travel time uncertainty is accumulated over the agent's trajectory.

If the travel time before entering e follows distribution $\mathcal{N}_{\text{time}}(\mu_{\triangleleft e}, \sigma^2_{\triangleleft e})$, the travel time just after finishing e would be

$$\mathcal{N}_{\text{time}}\left(\mu_{e\triangleright}, \sigma^2_{e\triangleright}\right) = \mathcal{N}_{\text{time}}\left(\mu_{\triangleleft e} + \mu_{\text{time}}(e, \pi), \sigma^2_{\triangleleft e} + \sigma_{\text{time}}(e, \pi)^2\right). \tag{S4}$$

This derives the lower and upper time bounds for when the agent in state s is on the target edge.

$$\begin{aligned} t_{\text{LB}}(s) &= \mu_{\triangleleft e(s)} - \alpha_{\text{time}} \cdot \sigma_{\triangleleft e(s)} \\ t_{\text{UB}}(s) &= \mu_{e(s)\triangleright} + \alpha_{\text{time}} \cdot \sigma_{e(s)\triangleright} \end{aligned} \tag{S5}$$

With these spatiotemporal uncertainty considerations, we now design a new state representation, $\langle t, e, \tau, \pi, \sigma_{e\triangleright} \rangle$, denoted as Ω_X. The first four are the same as in Ω_{hist}, while the last one represents the time uncertainty. The travel time T along e is based on the expectation of $\mathcal{N}_{\text{space}}$, i.e., $T(\pi, e) = \lceil \mu_{\text{time}}(\pi, e)/\delta \rceil$. The generation of the successor state follows Ω_{hist}, but when $\tau = T(\pi, e)$, a new state is generated as

$$\left\langle t + \delta, e', 1, \pi', \sqrt{\sigma^2_{e\triangleright} + \sigma_{\text{time}}(\pi', e')^2} \right\rangle. \tag{S6}$$

Note that, from the construction, $\sigma_{\triangleleft e}$ can be computed from the state information, i.e., $\sigma^2_{\triangleleft e} = \sigma^2_{e\triangleright} - \sigma_{\text{time}}(\pi, e)^2$. Similarly, $\mu_{\triangleleft e}$ and $\mu_{\triangleright e}$ can be recovered from t, τ, and $\mu_{\text{time}}(\pi, e)$. Consequently, a state $s \in \Omega_X$ has enough information to compute lower and upper time bounds in (S5). Together with the footprint Δ, we now have a binary and deterministic collision check function that takes motion uncertainty into account.

C.5 Problem formulation

Let us summarize the problem formulation of MAPF-X.

Definition 1. *An* MAPF-X instance *is defined by*

- *a geometric graph $G = (V, E)$, where each vertex lies in $\mathbb{R}^3$,*
- *a set of agents $A = \{1, 2, \ldots, n\}$,*
- *start $s_i \in V$ and goal $g_i \in V$ locations for each agent $i \in A$, and*
- *spatiotemporal motion model $\{\mu, \sigma\}_{\{\text{space,time}\}} : V^{N_{\text{MAPF}}} \times E \mapsto \mathbb{R}_{\geq 0}$,*

with hypermarameters of $\delta \in \mathbb{R}_{>0}$ as a unit of time, $N_{\text{MAPF}} \in \mathbb{N}_{\geq 0}$ which represents the length of the action history, and $\alpha_{\{\text{space,time}\}} \in \mathbb{R}_{\geq 0}$ which controls the conservatism of collision checking. We assume that the spatiotemporal motion model takes finite values.

Definition 2. *Given an MAPF-X instance, a state $s \in \Omega_X$ for an agent is a tuple consisting of*

- *$t \in [0, \delta, 2\delta, \ldots]$: time,*
- *$e = (v_{\text{from}}, v_{\text{to}}) \in E$: edge being traversed by the agent,*

- $\pi = (v_{-N_{\mathrm{MAPF}}}, v_{-N_{\mathrm{MAPF}}+1}, \ldots, v_{-1}) \in V^{N_{\mathrm{MAPF}}}$*: fixed-length waypoint history that the agent has recently visited,*
- $\tau \in [1, 2, \ldots, T]$*: progress index, corresponding to elapsed time on e, and*
- $\sigma \in \mathbb{R}_{>0}$*: standard deviation of the completion time of e.*

where $T = \lceil \mu_{\mathrm{time}}(\pi, e)/\delta \rceil$*. A* feasible *successor state* s' *of* s *is defined as*

- *when* $\tau < T$*:* $\langle t + \delta, e, \pi, \tau + 1, \sigma \rangle$
- *when* $\tau = T$*:* $\langle t+\delta, e', \pi', 1, \sigma' \rangle$*. Here,* $e' = (v_{\mathrm{to}}, \cdot) \in E$*,* $\pi' = (v_{N_{\mathrm{MAPF}}-1}, v_{N_{\mathrm{MAPF}}-2}, \ldots, v_{-1}, v_{\mathrm{from}})$*, and* $\sigma' = \sqrt{\sigma^2 + \sigma_{\mathrm{time}}(\pi', e')^2}$*.*

Definition 3. *Given an MAPF-X instance, an* MAPF-X problem *is to assign each agent i a feasible sequence of states,*

- *starting from* $\langle 0, (s_i, s_i), (s_i, \ldots, s_i), 1, 0 \rangle$*,*
- *ending at* $\langle \cdot, (\cdot, g_i), \cdot, T, \cdot \rangle$*, and*
- *without collisions between two states of different agents.*

Two states have a collision *when* (S1) *sufficies, where* Δ *and* $t_{\{\mathrm{LB,UB}\}}$ *are defined by* (S3) *and* (S5)*, repsectively. A* solution *is a list of paths for all agents that satisfies these conditions.*

For the optimization variant, we consider an accumulative cost minimization over *configurations*, which refer to a tuple of states for all agents, i.e., $Q \in \Omega_X^{|A|}$. Specifically, a cost for a solution $(Q_0, Q_1, \ldots, Q_m)$ is represented by $\sum_{k=1,\ldots,m} \mathtt{cost}(Q_{k-1}, Q_k)$, where `cost` takes non-negative values. For the implementation, we use

$$| \, i \in A \mid \neg\mathtt{isGoal}(Q_{k-1}[i]) \lor \neg\mathtt{isGoal}(Q_k[i]) | \tag{S7}$$

as the embodiment of `cost` following [24, 33]. A solution is called *optimal* if there are no other solutions that have a lower cost.

Remarks The above problem construction is inspired by advanced single- and multi-agent planning abstractions to adapt to physical robot constraints [63, 64, 43, 65, 44, 66]. Although these previous studies are typically evaluated in simplified simulations, our approach bridges the gap between theoretical frameworks and physical deployments through a carefully designed state space that reflects actual robot behavior and learning from robot trajectories. With this representation, the framework can generate kinodynamically feasible waypoints governed by physical constraints without explicitly modeling platform-specific state spaces. Another design consideration is the use of a discrete representation to efficiently apply combinatorial search algorithms. One might assume that the time deviation σ takes continuous values, but it will turn out that standard tree search algorithms can handle this in the next section.

D Solving MAPF-X

We are now interested in solving MAPF-X. Given a problem instance, an algorithm is *complete* if and only if it returns a solution in finite time for solvable instances; otherwise, it reports the non-existence of the solution. Furthermore, an algorithm is *optimal* if it always returns optimal solutions. The purpose of this section is to develop a complete and optimal algorithm for MAPF-X. We then describe our implementation, which is based on theory and practical considerations.

D.1 Basic tree search

The first step is to see that MAPF-X can be solved by a basic tree search algorithm. This is because, we can list all successor configurations given another $Q \in \Omega_X^{|A|}$. Let `getSuccessors` be such an enumeration. Then, Algorithm 1 solves MAPF-X in a complete and optimal manner.

Algorithm 1 Tree Search

1: $Open \leftarrow \emptyset,\ Goals \leftarrow \emptyset$
2: $Open$.append($\langle\, parent = \emptyset, config = Q_{\text{init}} \,\rangle$)
3: **while** $Open \neq \emptyset \wedge \neg$interrupted() **do**
4:     $N \leftarrow Open$.pop()
5:     **if** isGoal(Q) **then**
6:         $Goals$.append(N); **continue**
7:     **for** $Q_{\text{new}} \in$ getSuccessors($N.Q$) **do**
8:         **if** $\neg$duplicated(Q_{new}) **then**
9:             $Open$.append($\langle\, parent = N, config = Q_{\text{new}} \,\rangle$)
10: **if** $Goals \neq \emptyset$ **then**
11:     **return** backtrack$\left(\operatorname*{argmin}_{N \in Goals} \texttt{cost}(N)\right)$
12: **else**
13:     **return** NO_SOLUTION

This is actually an abstraction of the general search scheme, such as breadth-first search, depth-first search, or A*. Starting from the initial configuration Q_{init}, Algorithm 1 explores the tree structure of configurations by maintaining an *Open* list. *Open* stores a search node, a tuple of configuration and parent search node, which could be implemented by a stack, queue, or priority queue. The search process is finished when *Open* becomes empty. Then we can construct a solution by backtracking from the best goal node.

The idea is simple, but there is an important detail at Line 8 that prevents the search tree from growing infinitely; otherwise, the search cannot end in finite time because Ω_X is infinite due to the time t and the deviation σ, which can grow infinitely. We define duplicate detection as follows.

Definition 4. *A configuration Q is duplicated by Q' if one of the two conditions is true.*

- *All elements in Q and Q' are identical.*
- *Q' is an ancestor of Q in the search tree, and for each agent, $e \in E$, $\pi \in V^{N_{\text{MAPF}}}$, $\tau \in \mathbb{N}_{>0}$ are identical in Q and Q'.*

A function `duplicated`(Q) *returns* `TRUE` *if such a configuration* Q' *is in the search tree, otherwise returns* `FALSE`*.*

With this duplicated detection, we can derive completeness and optimality.

Theorem 1. *Algorithm 1 is complete and optimal for MAPF-X.*

Proof. The search finishes in finite time because the search tree does not infinitely grow. To see this, suppose contrary that the search never ends. This means that there is a path of configurations in the tree of infinite length, starting from Q_{init}, denoted as Π. Pick one configuraiton Q from Π. Due to the duplicate detection, the descendants of Q in Π never contain another configuraiton Q' such that, for each agent, $e \in E$, $\pi \in V^{N_{\text{MAPF}}}$, and $\tau \in \mathbb{N}_{>0}$ are identical in Q and Q'. Recall that since the spatiotemporal motion model takes finite values, the domain of τ is finite. Then the absence of such Q' in Π is impossible, since the combination of these three is finite. This concludes that the search finishes in finite time.

The duplicated node Q' with Q never contributes to the solution discovery. This is because the existence of a path Π that ends at a goal node that contains Q' indicates that Q can reach another shallow goal. Therefore, pruning Q' does not affect the completeness.

Due to its exhaustive nature, Algorithm 1 finds a solution if solutions exist, otherwise, it reports the non-existence. Moreover, the final outcome is selected from all possible solutions, resulting in an optimal solution. □

D.2 Tree-LaCAM

In practice, Algorithm 1 only works when the number of agents is very small. This is due to the exponential growth of the number of successors, called the branching factor. In other words, it becomes technically impossible to implement `getSuccessors`, which enumerates all successor configurations. The LaCAM algorithm (lazy constraints addition search) [32] provides a relaxation of this huge branching factor problem. Extended from Algorithm 1, the following introduces Tree-LaCAM, an adaptation of the original LaCAM for classic MAPF, those framed with Ω_{base}, to MAPF-X captured by Ω_X.

Algorithm 2 provides a simplified pseudocode of Tree-LaCAM, while gray-outinting the same procedures with Algorithm 1. The core idea is that instead of enumerating all successors at once, LaCAM generates each successor sequentially. This is achieved by configuration generation under constraints, which is implemented by adapting other MAPF algorithms, such as PIBT [22]. In the pseudocode, the variable C embeds a set of constraints that specify which agents should take which states, jointly with their ancestors. Line 13 then generates a configuration, while following constraints posed by C. A successor node will only be created if such a configuration is created and there are no duplicates in the search tree. The constraint structure is maintained within each search node, with the data structure of *tree*, assuming a queue. This can be understood as each node also performs a breadth-first search over constraints, which is called low-level search in the literature.

Theorem 2. *Tree-LaCAM (Algorithm 2) is complete and optimal for MAPF-X.*

Proof. The proof follows Theorem 1 and the completeness proof in [32].

Observe that each search node N is eventually discarded because, the number of agents and the number of successor states for each agent are finite, therefore $N.tree$ eventually becomes empty.

Algorithm 2 Tree-LaCAM

```
preface: C_init = ⟨parent = ∅, who = 0, state = ⊥⟩
Open ← ∅,  Goals ← ∅
Open.append(⟨ parent : ∅, config : Q_init, tree : [C_init]⟩)
while Open ≠ ∅ ∧ ¬interrupted() do
    N ← Open.top()
    if N.tree = ∅ then
        Open.pop(); continue
    if isGoal(Q) then
        Open.pop(); Goals.append(N); continue
    C ← N.tree.pop()
    if i = C.who + 1 ∈ A then
        for s ∈ getSingleAgentSuccessors(N.Q[i]) do
            N.tree.append (⟨ parent = C, who = i, state = s ⟩)
    Q_new ← configurationGenerator(N, C)
    if ∃Q_new ∧ ¬duplicated(Q_new) then
        Open.append(⟨ parent = N, config = Q_new, tree = [C_init] ⟩)
if Goals ≠ ∅ then
    return backtrack( argmin_{N∈Goals} cost(N) )
else
    return NO_SOLUTION
```

When this happens, all successor configurations from N have been added to *Open* due to the way constraints are constructed. Meanwhile, the search tree over configurations does not grow infinitely according to the discussion in Theorem 1. Consequently, Algorithm 2 conducts an exhaustive search for configurations that are reachable from Q_{init}, deriving the claim. □

In practice, Tree-LaCAM acts as an *anytime* algorithm, so that after initial solutions are found, solutions are refined over time and eventually converge to optimal solutions. Users can interrupt the refinement process at any time to obtain solutions, and in our usage, this is specified by the allotted offline planning time (e.g., 1-3 seconds).

Difference from the original A notable difference is the change in the search space representation, where the original LaCAM employs a search over configurations represented by a graph, while the above assumes a tree-shaped search space. This looks subtle, but is actually important for MAPF-X, which needs the notion of time in the state representation due to the propagation of time uncertainty. Meanwhile, the adaptation of tree representation raises another problem, that of the infinite growth of the search space. One implication of the analysis presented is that such growth can be prevented with appropriate duplicate detection, thus keeping the search space finite.

D.3 Implementation

Our implementation of Tree-LaCAM, which underlies all the demonstrations presented in this paper, is written in C++. In line with the original LaCAM, configuration generation with constraints is

implemented with the adapted version of PIBT [22], a popular lightweight MAPF algorithm. We incorporate some implementation-level optimizations from the recent developments in MAPF to improve the quality of the final solution within the limited planning time. These include search node reinsertion [32], random restart [24], node pruning based on known solution cost [24, 94], space utilization optimization [95], and Monte Carlo successor generation [33]. In addition, our code uses multi-threading to run multiple planners with different hyperparameter configurations simultaneously and passes the best solution among them to subsequent procedures. These techniques still hold the above theoretical establishment.

Meanwhile, for engineering reasons, the following two techniques are introduced into the implementation, which could compromise the theoretical guarantees of completeness.

Pruning large spatial deviation Our search implementation prunes robot states with large spatial deviations above a certain threshold. This breaks the complete search structure, but stabilizes the fast solution finding by shrinking the search space.

Duplicate detection Our implementation uses duplicate detection with a stricter version of the second condition in Definition 4, so that it prunes a configuration Q' if there is another Q in the *entire* search tree such that $\texttt{cost}(Q') \geq \texttt{cost}(Q)$. This reduces the computational cost of detecting duplicates while still providing a reasonable approximation of the original condition, as we do not need to backtrace all ancestors. Meanwhile, this remains an approximation because there may be evil cases where successors of two configurations Q and Q' have different successors without one being a complete subset of the other, due to time deviation differences. If these two belong to the same path in the tree, the descendant will have a subset of the ancestor's successors as the time deviation along the path increases. This is the reason why pruning does not affect the complete search structure in Algorithm 1. As we have not observed such evil cases after exhaustive trials, we have adopted the easy-to-implement approximation.

Integration with motion models The spatiotemporal motion model $\{\mu, \sigma\}_{\{\text{space,time}\}}$ is implemented by neural networks learned from actual robot behavior, which will be described later. The learning process is done with PyTorch, but the integration with Tree-LaCAM is done with a series of raw matrix calculations using the learned weight parameters. These are written directly in C++ using the Eigen library. This choice is based on inference speed. We have tested other styles, including the PyTorch C++ API, but have converged on the adopted style for performance.

Remarks We actually started the development with suboptimal versions of conflict-based search [96, 97], a celebrated MAPF algorithm. However, due to its poor scalability and slow response time, severely compromising mission-liveness, we switched to the latest powerful algorithm, i.e., LaCAM. The novel problem formulation called MAPF-X, the theory of Tree-LaCAM and its efficient implementation together underpin our various demonstrations of large-scale robot deployment.

E Materials and methods

This section complements the technical details of the proposed framework, especially for platform-specific implementations as our framework deploys both aerial and ground robots. The description adopts the East-North-Up (ENU) coordinate system as the world reference frame.

Please note that we do our best to provide sufficient detail compactly, but due to the vast nature of our implementation, which covers both aerial and ground robots, some technical presentations may differ slightly from the description below, although the conceptual levels are the same. We will be publishing most of the code, so please refer to it for further details.

E.1 Nominal robot dynamics

The robot is framed as a nonlinear control system with $\dot{\mathbf{x}} = f_{\text{nominal}}(\mathbf{x}, \mathbf{u})$, where $\mathbf{x} \in \mathcal{X} \subset \mathbb{R}^n$ and $\mathbf{u} \in \mathcal{U} \subset \mathbb{R}^m$. $\mathcal{X}$ and $\mathcal{U}$ are the state and control spaces, respectively. We use f_{nominal} to denote the nominal dynamics derived from first-principles, to distinguish it from the learned counterparts. While certain state variables, such as position and velocity vectors, are common to both aerial and ground robot platforms, other components can be platform-specific, such as angular state information; thus both n and m vary according to the platform under consideration.

Ground robot Our ground robot dynamics is abstracted as a linear system given that it is a holonomic robot. Specifically, the system equation is

$$\dot{\mathbf{x}} = \begin{bmatrix} \dot{p}_e \\ \dot{p}_n \\ \dot{v}_e \\ \dot{v}_n \\ \dot{\Theta} \\ \dot{\omega} \end{bmatrix} = \begin{bmatrix} 0 & 0 & 1 & 0 & 0 & 0 \\ 0 & 0 & 0 & 1 & 0 & 0 \\ 0 & 0 & 0 & 0 & 0 & 0 \\ 0 & 0 & 0 & 0 & 0 & 0 \\ 0 & 0 & 0 & 0 & 0 & 1 \\ 0 & 0 & 0 & 0 & 0 & 0 \end{bmatrix} \begin{bmatrix} p_e \\ p_n \\ v_e \\ v_n \\ \Theta \\ \omega \end{bmatrix} + \begin{bmatrix} 0 & 0 & 0 \\ 0 & 0 & 0 \\ 1 & 0 & 0 \\ 0 & 1 & 0 \\ 0 & 0 & 0 \\ 0 & 0 & 1 \end{bmatrix} \begin{bmatrix} u_{a_e} \\ u_{a_n} \\ u_{a_\omega} \end{bmatrix}. \tag{S8}$$

We denote the positional state $\mathbf{p}$ as $[p_e, p_n]$, and the orientation with respect to the U axis as Θ. Their time derivatives, i.e., velocity, are denoted as $[v_e, v_n, \omega]$. The control inputs are linear accelerations $[u_{a_e}, u_{a_n}]$ and angular acceleration u_{a_ω}. Once a desired control acceleration is obtained, we can use inverse kinematics to convert the accelerations into wheel speeds to steer the ground robot:

$$\begin{bmatrix} \Omega_1 \\ \Omega_2 \\ \Omega_3 \\ \Omega_4 \end{bmatrix} = \frac{1}{r_{\text{wheel}}} \begin{bmatrix} 1 & 0 & -L \\ 0 & 1 & -L \\ -1 & 0 & -L \\ 0 & -1 & -L \end{bmatrix} \begin{bmatrix} \cos(\Theta) & \sin(\Theta) & 0 \\ -\sin(\Theta) & \cos(\Theta) & 0 \\ 0 & 0 & 1 \end{bmatrix} \begin{bmatrix} u_{a_e}\Delta t + v_e \\ u_{a_n}\Delta t + v_n \\ u_{a_\omega}\Delta t + \Theta \end{bmatrix}, \tag{S9}$$

where r_{wheel} is the wheel radius and L is the distance from the center to each wheel, respectively. $\triangle t$ is the time interval between two control updates, which is used to convert the acceleration command to the target velocity. Then $\Omega_{i \in \{1,2,3,4\}}$ is the wheel speed that will eventually be sent to the robot's motor controller.

Quadrotor Let us define the rotation matrix that transforms forces from the quadrotor's body frame to the inertial frame:

$$\mathbf{R} = \begin{bmatrix} \cos\theta\cos\psi & \cos\theta\sin\psi & -\sin\theta \\ \sin\phi\sin\theta\cos\psi - \cos\phi\sin\psi & \sin\phi\sin\theta\sin\psi + \cos\phi\cos\psi & \sin\phi\cos\theta \\ \cos\phi\sin\theta\cos\psi + \sin\phi\sin\psi & \cos\phi\sin\theta\sin\psi - \sin\phi\cos\psi & \cos\phi\cos\theta \end{bmatrix}, \tag{S10}$$

where $[\theta, \phi, \psi]$ denotes roll, pitch, and yaw angle to the quadrotor's body axis. The full dynamical system for the quadrotor can be expressed as a function of the rotation matrix $\mathbf{R}$ [98]:

$$\dot{\mathbf{x}} = \begin{bmatrix} \dot{p}_e \\ \dot{p}_n \\ \dot{p}_u \\ \dot{v}_e \\ \dot{v}_n \\ \dot{v}_u \\ \dot{\phi} \\ \dot{\theta} \\ \dot{\psi} \end{bmatrix} = \begin{bmatrix} v_e \\ v_n \\ v_u \\ \mathbf{R}\begin{bmatrix} 0 \\ 0 \\ u_{\mathrm{T}} \end{bmatrix} + \begin{bmatrix} 0 \\ 0 \\ -g \end{bmatrix} \\ (u_\phi - \phi)/\triangle t \\ (u_\theta - \theta)/\triangle t \\ u_{\dot{\psi}} \end{bmatrix}. \tag{S11}$$

We denote the positional state $\mathbf{p}$ for the quadrotor as $[p_e, p_n, p_u]$, velocity $\mathbf{v}$ as $[v_e, v_n, v_u]$, and orientation as $[\theta, \phi, \psi]$. Gravitational acceleration is denoted by g while $\triangle t$ is the prediction time interval. The quadrotor's control inputs are target roll u_θ, pitch u_ϕ, yaw rate $u_{\dot{\psi}}$, and thrust u_{T}. These control commands are sent to the onboard flight controller.

E.2 Nonlinear model predictive control

With the forth-order Runge-Kutta integration scheme RK4, given the current robot state $\mathbf{x}_{\mathrm{current}} \in \mathcal{X}$, prediction horizon $N_{\mathrm{MPC}} \in \mathbb{N}_{>0}$, and the reference state trajectory $\mathbf{x}_{\mathrm{ref}} \in \mathbb{R}^{n \times N_{\mathrm{MPC}}}$ to be tracked, the MPC is formulated as a nonlinear optimization problem,

$$\begin{aligned} \min_{\mathbf{x}_k, \mathbf{u}_k} \quad & \sum_{k=0}^{N_{\mathrm{MPC}}-1} J(\mathbf{x}_k, \mathbf{x}_{\mathrm{ref}}, \mathbf{u}_k) \\ \text{subject to:} \quad & \mathbf{x}_{k+1} = \mathrm{RK4}\left(f_{\mathrm{nominal}}, \mathbf{x}_k, \mathbf{u}_k, \triangle t\right), \quad \mathbf{x}_0 = \mathbf{x}_{\mathrm{current}} \\ & g_{\mathrm{platform}}(\mathbf{x}, \mathbf{u}) \geq 0 \end{aligned} \tag{S12}$$

with the cost function defined as

$$J = \left[(\mathbf{x}_k - \mathbf{x}_{\mathrm{ref},k})^\top Q (\mathbf{x}_k - \mathbf{x}_{\mathrm{ref},k}) + \mathbf{u}_k^\top R \mathbf{u}_k\right] + (\mathbf{x}_{N_{\mathrm{MPC}}} - \mathbf{x}_{\mathrm{ref},N_{\mathrm{MPC}}})^\top P (\mathbf{x}_{N_{\mathrm{MPC}}} - \mathbf{x}_{\mathrm{ref},N_{\mathrm{MPC}}}), \tag{S13}$$

where $Q \in \mathbb{R}^{n \times n}$ is a state weighting matrix, $R \in \mathbb{R}^{m \times m}$ is for control, and $P \in \mathbb{R}^{n \times n}$ is for a terminal state. Note that all weight matrices are positive-definite. Both Q, R, and P are experimentally tuned to achieve the best navigation performance. The first control variable $\mathbf{u}_0$ is used as the control command. The platform-specific constraints g_{platform} will be described later, along with the trajectory optimization process. The implementation uses ACADOS [99] for real-time computation.

E.3 Discrete-time control barrier function (D-CBF)

The framework employs D-CBF to enforce safety by preventing inter-agent collisions and collisions with other obstacles. Formally, we define the safety set $C = \{\mathbf{x} \in \mathcal{X} \mid h(\mathbf{x}) \geq 0\}$, where:

- $h(\mathbf{x}) : \mathbb{R}^n \to \mathbb{R}$ is a continuously differentiable function that defines the safety set.
- $h(\mathbf{x}_k) \geq 0$ ensures that the state $\mathbf{x}_k$ remains within the safe set at step k.

With its time derivative $\dot{h}$ and a hyperparameter $\gamma \in \mathbb{R}_{>0}$ that governs the conservativeness of safety, the discrete CBF constraint can be written as:

$$\dot{h}\left(\mathbf{x}_k, \mathbf{u}_k\right) + \gamma h\left(\mathbf{x}_k\right) \geq 0 \tag{S14}$$

The system will remain in a safe state unless this constraint is violated. See [36] for the mathematical background.

Our implementation defines safe states assuming ellipsoidal rigid bodies, i.e,

$$h(\mathbf{x}) = 1 - \frac{(p_e - p_{e,c})^2}{a^2} + \frac{(p_n - p_{n,c})^2}{b^2} + \frac{(p_u - p_{u,c})^2}{c^2} \tag{S15}$$

where a, b, c defines the safety distance in the corresponding coordinates, and $[p_{e,c}, p_{n,c}, p_{u,c}]$ the centroid of the object. The derived CBF constraint is added to the MPC formulation (S12) to enforce safety for all robots [37]. In practice, obstacle observation limits based on robot positions are introduced to avoid imposing unnecessary constraints.

Backup controller CBF constraints can provide safety guarantees, but in practice, especially in crowded situations with multiple robots, MPC often falls into an infeasible configuration. In this case, the framework uses a backup controller, a strategy often applied to controllers with aggressive maneuvers [100]. Specifically, instead of imposing CBF constraints, the backup controller sets $\mathbf{x}_{\text{ref}}$ to $\mathbf{p}_{\text{current}}$ and then solves the MPC. This process encourages the robot to stay at its current position with its best effort, resulting in safer behavior synthesis.

E.4 Learning based system identification

During the trajectory optimization, the framework uses a gray-box dynamics representation f_{gray}, which is a summation of the nominal dynamics f_{nominal} and the residual dynamics f_{NODE} learned from the actual robot trajectories. f_{NODE} is represented as neural ordinary differential equations (NODE) [34]. In both ground and aerial robots, the implementation uses four-layer neural networks with 64 neurons in each hidden layer. Instead of predicting the full robot state, NODE is introduced for state variables where the prediction error by f_{nominal} is large, after inspecting the collected data: $[p_e, p_n, \Theta]$ for the ground robot, and $[\theta, \phi, \psi]$ for the quadrotor. This reduced complexity helps to solve trajectory optimizations stably. Training is provided using the Torchdyn library [101] with over 50,000 data points consisting of over $1,000$ seconds of robot motion for each platform. Data collection is performed using MPC tracking on specially designed trajectories to cover the target robot states. An example trajectory is visualized in Figure 8H.

E.5 Reference trajectory via numerical optimization

Given a 'dense' waypoint sequence $\left[\mathbf{p}'_1, \mathbf{p}'_2, \ldots, \right] \in \mathbb{R}^{3\times N_{\text{traj}}}$, initial state $\mathbf{x}_{\text{init}} \in \mathcal{X}$, terminal states $X_{\text{fin}} \subset \mathcal{X}$, and deviation tolerance $\overline{d}_{\text{traj}} \in \mathbb{R}_{\geq 0}$, we formulate the trajectory optimization using N_{traj} state and control variables associated with the sampling time, over the gray-box dynamics f_{gray}:

$$
\begin{aligned}
\min_{\mathbf{x}_k, \mathbf{u}_k, \triangle t_k} \quad & \sum_{k=0}^{N_{\text{traj}}-1} \triangle t_k \\
\text{subject to:} \quad & \mathbf{x}_{k+1} = \mathtt{RK4}\left(f_{\text{gray}}, \mathbf{x}_k, \mathbf{u}_k, \triangle t_k\right), \quad \mathbf{x}_0 = \mathbf{x}_{\text{init}}, \quad \mathbf{x}_{N_{\text{traj}}-1} \in X_{\text{fin}}, \\
& \left\|\mathbf{p}'_k - \mathbf{p}_k\right\| \leq \overline{d}_{\text{traj}} \\
& g_{\text{platform}}(\mathbf{x}_k, \mathbf{u}_k) \geq 0
\end{aligned}
\tag{S16}
$$

$\mathbf{p}_k$ is a positional state in $\mathbf{x}_k$. The formulation forces the resulting trajectory not to deviate too much from the given waypoint sequences, while still minimizing the arrival time.

This trajectory optimization is a multiple shooting method, where both the state variables $\mathbf{x}$ and control variables $\mathbf{u}$ are treated as decision variables over the discrete time horizon, and then additional equality constraints are added to ensure that the state trajectory satisfies the system dynamics between neighboring time points. The multiple shooting provides better numerical stability for highly nonlinear systems and yields high-quality training datasets for the learning components. The implementation is based on IPOPT with CasADi libraries, which differs from MPC due to their representational flexibility.

E.6 Platform-specific constraints

Based on our prior knowledge of each robot platform, the framework imposes platform-specific constraints g_{platform} on both the MPC (S12) and offline trajectory optimization (S16) processes. Since (S12) and (S16) have similar forms, some platform-specific constraints are common between them. Meanwhile, the implementation of MPC removes several constraints from those of trajectory optimization due to the need for real-time response and the kinodynamic awareness provided by the reference trajectory.

Ground robot For each state and control variable, $\mathbf{x}_k$ and $\mathbf{u}_k$, we impose the following constraints on the trajectory optimization of the ground robot.

$$
\|\mathbf{v}_k\| \leq v_{\max}, \quad \left\|[a_{e,k}, a_{n,k}]\right\| \leq a_{\max}, \quad |\omega_k| \leq \omega_{\max} \tag{S17}
$$

$$
\left|v_{e,k}\cos(\Theta_k) + v_{n,k}\sin(\Theta_k)\right| \leq v_{\max,\text{lateral}} \tag{S18}
$$

$$
\frac{\|\mathbf{v}_k\|^2}{R_k} \leq c_{\text{f,limit}} \tag{S19}
$$

The first line limits the speed and acceleration, while the second one imposes the lateral speed bound aligned with the robot's ego frame. To avoid trajectory overshooting during the deployment, we also add (S19), which approximates the centripetal force [102] with the circumcircle radius R_k, computed by:

$$
R_k = \frac{\|\mathbf{p}_{k-1} - \mathbf{p}_{k+1}\|}{2\sin\left(\arccos\left(\frac{(\mathbf{p}_{k-1}-\mathbf{p}_k)^\top(\mathbf{p}_{k+1}-\mathbf{p}_k)}{\|\mathbf{p}_{k-1}-\mathbf{p}_k\|\cdot\|\mathbf{p}_{k+1}-\mathbf{p}_k\|}\right)\right)} \tag{S20}
$$

MPC only uses (S17).

Quadrotor The quadrotor-specific constraints for trajectory optimization consist of:

$$\left|u_{\theta,k}\right| \leq u_{\theta,\max}, \quad \left|u_{\phi,k}\right| \leq u_{\phi,\max}, \quad \left|u_{\psi,k}\right| \leq u_{\psi,\max}, \quad 0 \leq |u_T| \leq u_{T,\max} \tag{S21}$$

$$|\theta_k| \leq \theta_{\max}, \quad |\phi_k| \leq \phi_{\max}, \quad |\psi_k| \leq \psi_{\max} \tag{S22}$$

$$\|(\mathbf{v}_{k+1} - \mathbf{v}_k) / \triangle t_k\| \leq a_{\max} \tag{S23}$$

$$p_u \geq 0 \tag{S24}$$

The first line limits the control commands, while the second line corresponds to the attitude constraints, limiting the maximum roll, pitch, and yaw for flight stability. The third line limits the acceleration, the threshold value of which has been determined through iterative flight testing by gradually increasing this limit until flight instability occurs. We enforce a height constraint at the end. MPC only uses (S21).

E.7 Reference trajectory generation via transformers

To reduce the computational overhead, the framework generates reference trajectories via imitation learning inference instead of numerical optimization. Specifically, given a dense waypoint sequence $[\mathbf{p}'_1, \mathbf{p}'_2, \ldots,]$, we first compute a waypoint-wise feature for $\mathbf{p}'_k$ as concatenation of: (i) positional difference $\mathbf{p}'_{k-1} - \mathbf{p}'_k$ and $\mathbf{p}'_{k+1} - \mathbf{p}'_k$; and (ii) start and goal binary indicators whether $\mathbf{p}'_k$ is a start or goal location. Additionally, for the ground robot only, (iii) we include the angle information of the initial state x_{init} as $[\sin(\Theta_{\text{init}}), \cos(\Theta_{\text{init}})]$. These features are subsequently fed into a transformer-based [35] prediction model, the parameters of which are available in Table S2. The model directly predicts most of the state variables for each $\mathbf{p}'_k$, along with the time difference $\triangle t_k$ from the previous state. Meanwhile, the positional prediction uses the difference between $\mathbf{p}'_k$ and its groundtruth after the trajectory optimization, and angular information for the ground robot uses cos-sin encoding. The resulting state sequence is then parameterized with spline curve interpolation to match the sampling rate to other processes.

Training The training dataset for each platform was generated by numerical optimization (S16) on three computer clusters, using more than 300 CPU cores in total with extensive parallelization, over almost two full days. Waypoint sequences were generated by a random walk with densification of linear interpolation. This process yielded a dataset of over $100,000$ samples that can be used as groundtruth labels. The model training converged in approximately five hours using dual NVIDIA A100 GPUs. While the data generation phase demands significant computational resources, it is performed entirely offline, making the subsequent deployment computationally efficient during the deployment phase.

E.8 Spatiotemporal motion modeling

The MAPF-X problem uses the motion model that encodes the spatiotemporal tracking performance of real robots, represented by the Gaussian distribution $\mathcal{N}_{\{\text{space,time}\}}$. With a predefined geometric roadmap $G = (V, E)$, the conditioning is made by the robot action history,

Table S2: **Model architecture and parameters for reference trajectory generation.** These numbers are based on 57 waypoints input for ground robots. The input dimension is 11 (position: 4, start-goal flag: 2, angle: 2), including the 3 dimensions discarded in early development. The output dimension is also 11 (position: 2, velocity: 2, angle: 2, angular velocity: 1, time: 1), with control commands (3) which have not been used. The positional encoding is implemented as a concatenation form. The identical architecture is employed for the drone, but with different input and output layers to adapt its state space.

**Layer**	**Output shape**	**Param #**
Input	[57, 11]	
Linear	[57, 252]	3,024
PositionalEncoding		-
TransformerEncoderLayer	[57, 256]	395,776
TransformerEncoderLayer	[57, 256]	395,776
TransformerEncoderLayer	[57, 256]	395,776
TransformerEncoderLayer	[57, 256]	395,776
Linear	[57, 11]	2,827
Total params:		1,588,955

$(\mathbf{p}_{t-N_{\text{MAPF}}}, \mathbf{p}_{t-N_{\text{MAPF}}+1}, \ldots, \mathbf{p}_{t-1}) \in V^{N_{\text{MAPF}}}$, and the current traveling edge $(\mathbf{p}_t, \mathbf{p}_{t+1}) \in E$, collectively represeted as a waypoint trajectory $P_t = [\mathbf{p}_{t-N_{\text{MAPF}}}, \mathbf{p}_{t-N_{\text{MAPF}}+1}, \ldots, \mathbf{p}_{t-1}, \mathbf{p}_t, \mathbf{p}_{t+1}] \in \mathbb{R}^{3\times(N_{\text{MAPF}}+2)}$. N_{MAPF} is set to three during the experiments. Then $\mathcal{N}_{\{\text{space,time}\}}$ predicts the travel time and the spatial deviation when a robot is about to travel from one waypoint $\mathbf{p}_t$ to another $\mathbf{p}_{t+1}$.

The prediction is implemented as a three-layer neural network with 64 neurons in each hidden layer for both ground and aerial robots. The model takes P_t as input and outputs $\{\mu, \sigma\}_{\{\text{space,time}\}}$. P_t is pre-processed by normalizing each component with $\mathbf{p}_t$ before passing the vectors to q. We trained the network q with more than 50,000 samples collected from real robot trajectory tracking for each platform. The data collection took approximately four hours each.

E.9 Translation of timed waypoints to reference trajectory

A solution to MAPF-X for each robot is a sequence of states in Ω_X. We then extract the states where the progress index τ is one, resulting a spatiotemporal sequence $\mathcal{P} := [\langle \mathbf{p}_1, t_1 \rangle, \langle \mathbf{p}_2, t_2 \rangle, \ldots]$. There is a consideration to translate $\mathcal{P}$ to a state trajectory with the transformer because $\mathcal{P}$ may contain a 'wait' action $[\langle \mathbf{p}_k, t_k \rangle, \langle \mathbf{p}_{k+1}, t_{k+1} \rangle]$ where $\mathbf{p}_k = \mathbf{p}_{k+1}$. This waiting time, $t_{k+1} - t_k$, can vary depending on how the robots coordinate, and is difficult to handle in the learning-based trajectory inference. Therefore, the implementation first splits the original solution $\mathcal{P}$ into several segments $\mathcal{P}_1, \mathcal{P}_2, \ldots, \mathcal{P}_l$ according to the wait actions, and then performs the trajectory inference for each segment. The resulting trajectories are combined while inserting the wait times from the MAPF result. This combined trajectory is finally sent to the MPC as a reference trajectory.

E.10 Roadmap generation

In the experimental arena, the motion capture system is responsible for tracking all objects, including obstacles, in real-time. Based on the position information, the roadmap G is generated periodically (4 Hz) and asynchronously to feed the MAPF planner with the latest information. The implementation models all objects as ellipses, thus collision checking is conducted with simple geometric calculations. The roadmap is based on grid sampling, but its granularity, specified by the sampling step distance and the connection radius between two vertices, is adapted to each experimental scenario. As a reference, in our 40-robot demonstration, the roadmap contains about 600 vertices for the ground robots, with a step size of 0.2 m and a maximum connection radius of 8 m. The quadrotor case contains 700 vertices, with a step size of 0.3 m and a maximum connection radius of 2 m. The code is written in Python, and integrated into the GUIs. It should be noted that with many vertices, real-time roadmap generation and rendering can be challenging, a major factor in the use of the above numbers.

E.11 Deployment setups

Object tracking and communication The framework is deployed within a laboratory workspace measuring $7.0 \times 5.0 \times 1.8$ m^3 (Figure 2B). Object tracking is based on the OptiTrack motion capture system using 12 infrared cameras operating at 60 Hz to track a maximum of 40 objects in our largest-scale experiments. The inter-robot communication infrastructure relies on ROS 2 Data Distribution Service (DDS) middleware, with all devices connected through a central router. To ensure reliable operation at this scale, we have made extensive optimizations to communication protocols, control algorithms, and motion capture configurations. Particular attention has been paid to preventing network bandwidth saturation, which could potentially destabilize the entire robot fleet.

Computation As shown in Figure 2A, each ground robot is equipped with an NVIDIA Orin NX module, enabling onboard trajectory generation and MPC calculations. We utilize Docker containers to streamline the system setup across all units. For the aerial platforms, comprising 20 quadrotors, the high-level controllers are located offboard on a Mac Studio with M1 CPUs, due to limitations in onboard resources. To evaluate the robustness of the system, we introduce 12 robots based on TurtleBot3. These autonomous obstacles operate without user input, serving solely to increase environmental randomness and demonstrate our method's capability to handle scenarios not considered during the plan preparation phase.

Graphic user interface We design real-time GUIs, one for the ground robots and another for the quadrotors, to control the robot fleet interactively. These software are based on ROS 2 and Matplotlib libraries. During the experiments, two GUIs were run separately on MacBook Pros with M2/M3 Max CPUs. Figure 8A includes their appearance, where users can easily assign target positions to robots using drag-and-drop operations, and then launch a multi-robot navigation mission with a single button click. At the backend, each GUI instance is combined with the MAPF planner. The GUI also acts as a roadmap generator, periodically and asynchronously updating the roadmap used in MAPF based on feedback from the arena configuration. It is also equipped with various features such as roadmap graph sparsity specification, workspace constraints, autoland command

for quadrotors, ROS bag recording, and trajectory replay, to name just a few. This user-friendly and visual debugger underpins our comprehensive robot deployment.

E.12 Computational resource allocation and communication load

Often overlooked in simulation studies is the inherently distributed nature of multi-robot systems, which has practical implications on allocating computational resources in real-robot deployments and is closely tied to communications overhead. As the number of processes participating in the network increases, the reliability of communication is generally compromised due to packet loss occurrences and message delays, which could have catastrophic consequences for the execution of agile robot motion. In particular, our demonstration comprises a maximum of 40 robots, including both ground and aerial robots, which collectively place a significant load on the network infrastructure. Therefore, the implicit challenge was to optimize the resource allocation, which resulted in the configuration depicted in Figure 2C. Owing to the limitations of the hardware, the quadrotors are currently dependent on an offboard MPC controller. The decoupled controllers were parallelly executed on a single powerful computer, which also hosted a positional information broadcaster, to minimize the number of inter-router packets. Meanwhile, generating reference trajectories and solving the MPC optimization onboard transferred the computational burden away from the central component to the ground robots. This flexible allocation is feasible thanks to our modular approach, which permits the system to coexist with a central and potentially slow MAPF planner, and a distributed reactive controller while optimizing the communication stack. Collectively, these elements form the basis for the unprecedented demonstrations.

F Empirical observation of learning-based reference trajectory generation

We have introduced learnable components for reference trajectory generation because of the practical considerations of deploying many robots simultaneously with real-time response. Specifically, the framework uses two neural networks: Neural ODE (NODE) to better model robot dynamics, and a Transformer model to instantly approximate trajectory optimization.

To remark their necessity, in Figure S2, we compare quadrotor tracking performance, using reference trajectories obtained from trajectory optimization with nominal dynamics f_{nominal}, and with gray-box dynamics f_{gray} enhanced by NODE. Note that their optimization formulations are exactly the same, the only difference being the representation of the dynamics. As a whole, these two reference trajectories result in a subtle geometric difference, however, the tracking error is actually higher for f_{nominal} as evidenced in Figure S2(B). The trajectory with f_{nominal} assigns a time allocation that assumes too aggressive flights to be tracked, while the trajectory with f_{gray} moderately suppresses such an infeasible allocation.

Figure S2(C) illustrates this reason, where the state prediction with f_{nominal} causes large errors from the actual state in roll and pitch. More specifically, f_{nominal} can generate states closer to the step response than the actual ones, which accelerates the trajectory. A better dynamics representation than (S11) may reduce such error terms, e.g., those with inertia if known, as used in [51]. But instead, we take data-driven modeling that we can employ with our infrastructure. This practical

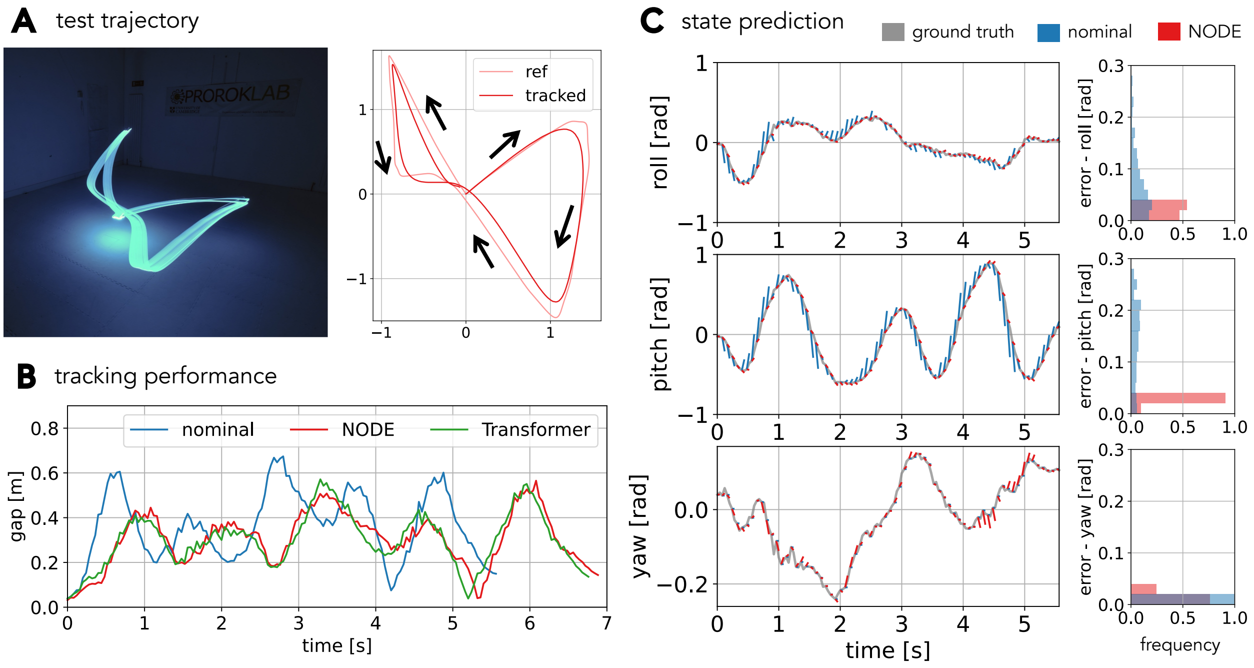

Figure S2: **Example of reference trajectory tracking. (A)** Light trajectory image of the test case and top-down view of reference and tracked trajectories. **(B)** Tracking gap evaluated with $\|p(\mathbf{x}_{\mathrm{ref},0}) - p(\mathbf{x}_{\mathrm{current}})\|$, where $p(\mathbf{x})$ is the position vector of state $\mathbf{x}$. The references are generated with trajectory optimization based on either f_{nominal} or f_{gray} (i.e., with NODE); other parameters are identical. We also tested the transformer, learned from f_{gray}. NODE suppresses unacceptable aggressive behavior and the transformer follows suit. **(C)** State prediction of rotational components from nominal and NODE dynamics against ground truth from **(A)**. While the prediction errors of other components (i.e., position and velocity) are moderate, we observed significant errors in roll and pitch with (S11), leading us to introduce the gray-box dynamics modeling.

consideration results in learning residual dynamics with NODE, which efficiently reduces state prediction errors, and thus reduces the tracking errors as well. These improvements may seem small, but they are critical for handling large numbers of robots while still allowing for agile movement, as the probability of failure in multi-robot systems increases exponentially with the number of robots.

The introduction of the transformer is another practical consideration for the real-time aspect. Even with state-of-the-art methods [52, 53] that use extensive knowledge of well-studied system dynamics, time-optimal trajectory optimization takes a non-negligible amount of time. Meanwhile, we need to complete this process immediately for many robots, without any significant time difference. This justifies the approximation of trajectory generation with imitation learning, i.e., the transformer, which can generate reference trajectories in sub-milliseconds, even with onboard computing resources. As seen in Figure S2(B), well-trained transformers capture the characteristics of the generated trajectories with f_{gray}, resulting in similar tracking performance even in unforeseen cases. The artifact used in the framework was chosen according to these empirical results.

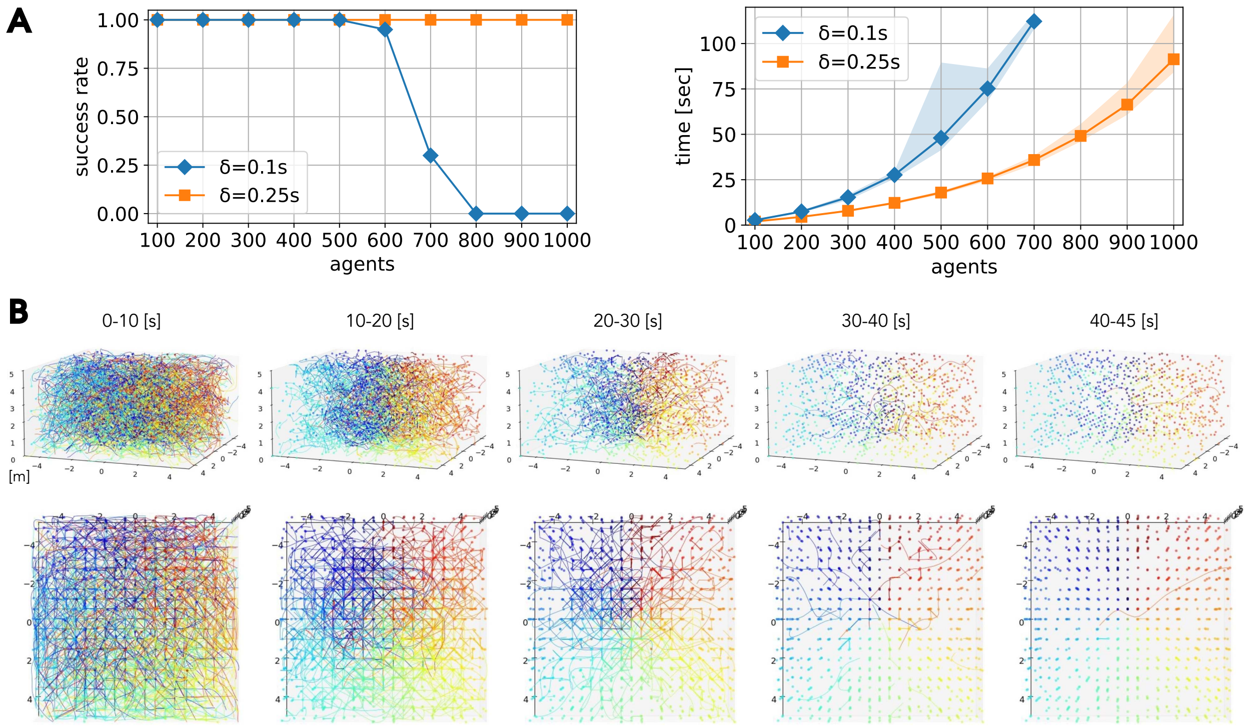

Figure S3: **Scalability test.** This experiment is conducted using the parameters used in Figure 3, within $10 \times\ 10 \times\ 5\text{m}^3$ empty space, on a roadmap where vertices are placed for each 0.5m with a connection radius of 1.5m. **(A)** Success rate of the concrete planning within 2 minutes timeout, and runtime to find initial feasible solutions. Scores are calculated over 20 instances with randomized start and target locations. The semi-transparent areas represent the minimum and maximum runtime. The computational load of the planning problem is affected by how finely the time is discretized, which is specified by δ in MAPF-X. Note that a finer δ would better reflect real robot behavior. **(B)** Trajectory visualization for a $1,000$ quadrotor case, generated by the transformer based on the MAPF plan with $\delta = 0.25$s.

G Scalability assessment for MAPF-X planner

Figure 7E assesses the planner's scalability, while this section provides further insight, but with hundreds of robots. Figure S3(A) summarizes the planning stress test, using the same computing environment as the demonstrations, while changing δ, a unit of time in MAPF-X, over 20 randomized scenarios within a larger empty space. This time, Tree-LaCAM does not use the space utilization optimization, as it is known to be time-consuming for large instances [33, 103].

The results show that even with hundreds of agents, the current implementation can derive feasible and full horizon solutions from start to goals, given sufficient time of tens of seconds. Coarsening the planning time interval δ further improves real-time planning capability and hence scalability. With $\delta = 0.25$s, our implementation can handle a thousand quadrotors. Example trajectories are visualized in Figure S3(B). This speedup is due to the reduction in the number of collision checks with a coarse time unit.

At this scale, the main bottleneck is collision checking, where we need to ensure that there are

no collisions between any pair of states from any pair of agents. Imposing geometric constraints on edge assumptions can reduce this exhaustive check, whereas ours currently accepts arbitrary roadmap structures to maintain generality. Since the original LaCAM for grid-world can achieve pathfinding for ten thousand agents in seconds [24], we believe that the pursuit of further scalability and real-time responsibility is feasible, but it will require such additional assumptions. Improving the quality of solutions for large swarms also remains a major challenge.

H Baseline implementations for the ground robots experiments

Figure 4 introduces two baseline methods, which are elaborated below. Both methods are categorized as a coupled, centralized approach. We have also tested fully decentralized approaches, as mentioned in the ablation study section, but these have not resulted in successful navigation trials.

H.1 Grid-world planning followed by ADG execution

This method first solves MAPF under the assumption of a grid-world roadmap representation, while ignoring kinodynamics. Then, during execution, a central scheduler sends wait-or-go commands to each robot following the MAPF plan, without breaking the temporal dependencies of who will visit the same location earlier than another. Doing so ensures deadlock-free execution of the pre-computed plan. This temporal dependency is represented as a graph structure between agent actions, hence it is called Action Dependency Graph (ADG) [40, 104, 41], also known as Temporal Plan Graph (TPG). ADG is a popular method to implement MAPF systems to enforce the safe execution [105, 42, 106]. However, it assumes simple collision checking, which is typically implemented with grid-world planning; otherwise, the construction could be a time-consuming process [92], and thus not suitable for our dynamic environments.

The MAPF plan is prepared by LaCAM* without the learned dynamics. The low-level controller is implemented by a combination of trajectory generation with a trapezoidal velocity profile, and MPC tracking without CBF. The experiments include several inter-robot collisions, as we set the low-level controllers to perform aggressive movements that may result in spatial deviations from the plan, to be competitive with the performance of the proposed method. We could set conservative motions, but such a configuration would result in poorer navigation performance, as longer navigation times mean there is a high chance that moving obstacles will block the robots' progress. We also note that the four-connected grid-world representation narrows the available space for robots, thus increasing the chance that robots close in on each other. Nevertheless, we had to cut off the CBF in order to get a successful navigation sequence, as they could trigger deadlock situations due to the high-level planner's assumption of too-close coordination. This could be mitigated by increasing the grid size, but then we often encountered infeasible MAPF instances due to the stochastic motions of dynamic obstacles. In short, ADG is nuanced in dynamic environments, and we employed the stable implementation configuration for the long-term mission.

H.2 Any-angle planning followed by naive execution

This method allows the robot to move in any direction between two points on the grid, and is therefore no longer limited by mere geo-adjacent relationships. Technically, such problems are

called any-angle path planning and were originally developed for single-agent scenarios [107, 108]. Recent studies have extended this notion to multi-agent scenarios [43, 109], or, more generally, to arbitrary graph representations embedded in geometric space or arbitrary travel time assumptions to derive smoother coordination [65, 44, 45, 110, 111, 112, 92]. For the sake of simplicity, we refer to these scenarios collectively as any-angle planning.

Observe that our MAPF planner has already been categorized into such a planning style, as it allows for arbitrary roadmap representations. We, therefore, implement any-angle MAPF directly with LaCAM*, but without kinodynamic consideration. Instead, collision checking is based on distance traveled, assuming that each robot can move at a constant speed with no other speed profiles, as is often assumed in simulation-based MAPF studies. These high-level plans are tracked by the same low-level controller as used in ADG.

As shown in Figure 4, the resulting trajectories contain many collisions. This is due to the mismatch between the kinodynamic agnostic high-level planner and the low-level controller, which simply tries to follow the high-level plans as best it can. Note that, as in the discussion with ADG, we can reduce this mismatch by reducing the agility of the robot's movements, but such schemes will not only reduce navigation performance, but will also trigger another problem with dynamic obstacles in our environments due to the longer mission completion time. Similarly, introducing CBF could mitigate collisions, but alternatively introduce deadlock situations.

I Juxtaposing with decentralized quadrotor control

Our work is not specific to quadrotor swarm trajectory generation, but rather is a general scheme for solving multi-robot motion planning with high transferability across different robot platforms. Meanwhile, especially in the multi-drone community, considerable research has been devoted to establishing decoupled planning, amenable to decentralized deployments, which are different axes towards multi-robot coordination. Both approaches have their own advantages and disadvantages, have different areas of application, and should therefore be developed. Nevertheless, it is useful to juxtapose the two in order to highlight their nature, and this section provides such insights.

I.1 General discussion

As a general explanation, decentralization, or decoupled planning, aims to make coordinated behavior 'emerge,' while coupled planning 'designs' coordination. As is often discussed, the gain in computational efficiency that comes with real-time adaptivity comes at the expense of guaranteed coordination. This makes decoupled planners unsuitable for tight and dense scenarios such as warehouse automation. In fact, to the authors' knowledge, some leading industrial companies have adopted coupled planners. On the other hand, quadrotors often require only 'sparse' coordination because their workspace is usually much larger than their footprint; deadlock or livelock events are mitigated or completely resolved by timing randomness. This leads to successful decentralization developments in quadrotor swarm control.

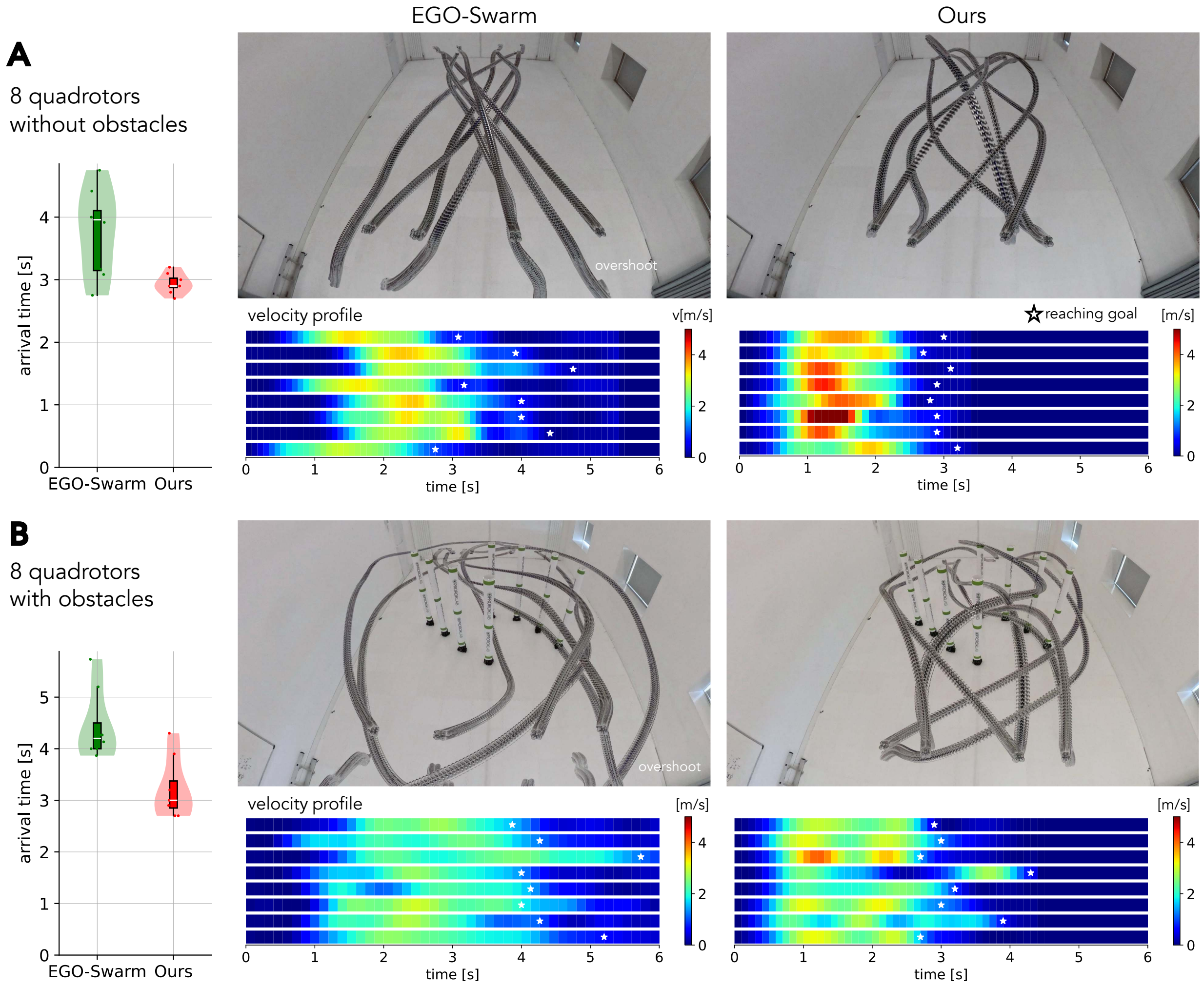

Figure S4: **Trajectories generated by a decentralized baseline method and ours.** In addition to the proposal, we deployed actual quadrotors using EGO-Swarm [39], a representative decentralized method for quadrotor swarm trajectory generation. Its implementation is the one provided by the authors with a ROS 2 integration. The tasks are to swap their positions, with one group on one side and another group on the other, in **(A)** an empty space, and **(B)** a workspace containing obstacles. EGO-Swarm's parameters, such as maximum speed and acceleration, are adjusted to maximize agility while allowing safe navigation for the respective scenario; at higher speeds, the quadrotors experienced multiple collisions. The figure shows the arrival time near the destination, timelapse trajectories, and velocity profiles. We note that, in the scenario **(A)** with our framework, one quadrotor reaches 6.5m/s.

I.2 Aggressive coordination

Another notable difference is the ability to coordinate aggressively. Figure S4 (partially presented in Figure 3E) and Movie S6 provide empirical evidence with eight quadrotors deployed, using an

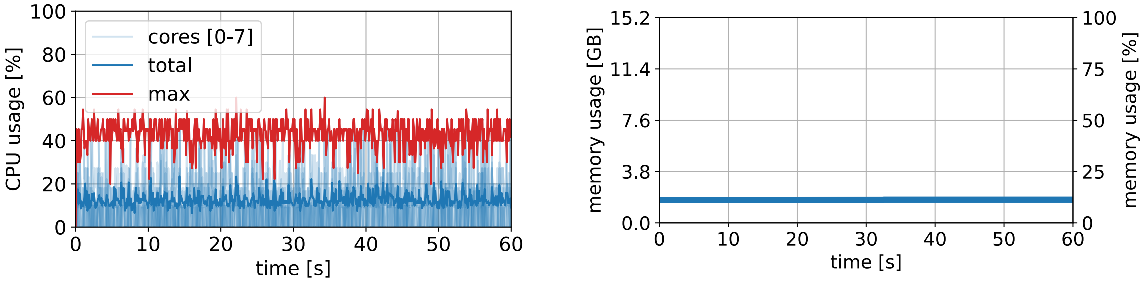

Figure S5: **Onboard CPU and memory usage.** The data was collected by deploying four ground robots with four static obstacles over 1 minute of repeated missions. We have confirmed that CPU and memory usage are similar regardless of the number of robots and obstacles used. GPUs are not used during deployment.

established decentralized planner called EGO-Swarm [39].[1] Its parameters have been adjusted to be competitive with ours in terms of flight speed. Two scenarios, without or with obstacles, are prepared, which have almost identical start and goal positions.

First, we can see the difference in robot-wise mission completion time. This difference is partly due to the platform-aware time-optimal trajectory generation used in the proposed method, which extracts the hardware capability. But the fundamental difference is that decentralized methods have to be conservative to avoid collisions. The velocity profile in Figure S4(A) well illustrates this phenomenon, where EGO-Swarm poses some quadrotors at their starting points when the mission begins, while all quadrotors simultaneously depart from their starting points and immediately reach high speed in our coupled approach. The scenario in Figure S4(B) also shows that EGO-Swarm assigns longer trajectories to be safe, because it cannot discover tightly coupled trajectories due to its planning representation. As a reference, in discrete domains, similar empirical evidence abounds showing that there are substantial performance gaps between centralized and decentralized planning outcomes, e.g., [113, 114, 25, 115]

We do not claim that coupled planning is better than its decoupled counterpart. In particular, there is a notable difference in terms of computational cost, as ours requires non-negligible preparation time. Rather, the observation here is that coupled planners can synthesize aggressive coordination that is difficult for decoupled planners. This motivates us to develop faster and more scalable coupled planners as the holy grail of research in this area.

J Onboard CPU and memory usage

For the ground robot, a robot-wise controller, consisting of a reference trajectory generator implemented by the transformer and MPC tracking, runs within the onboard computing resources, NVIDIA Jetson Orin board in our case. One possible question is how expensive they are. We provide empirical evidence here.

Figure S5 shows CPU and memory usage. Note that the transformer inference runs on the CPU, not the GPU. This is because the inference is not for a batch of queries, but for a single query at

[1]The implementation is from `https://github.com/ZJU-FAST-Lab/ego-planner-swarm`. We used the ROS2 version due to our deployment environment.

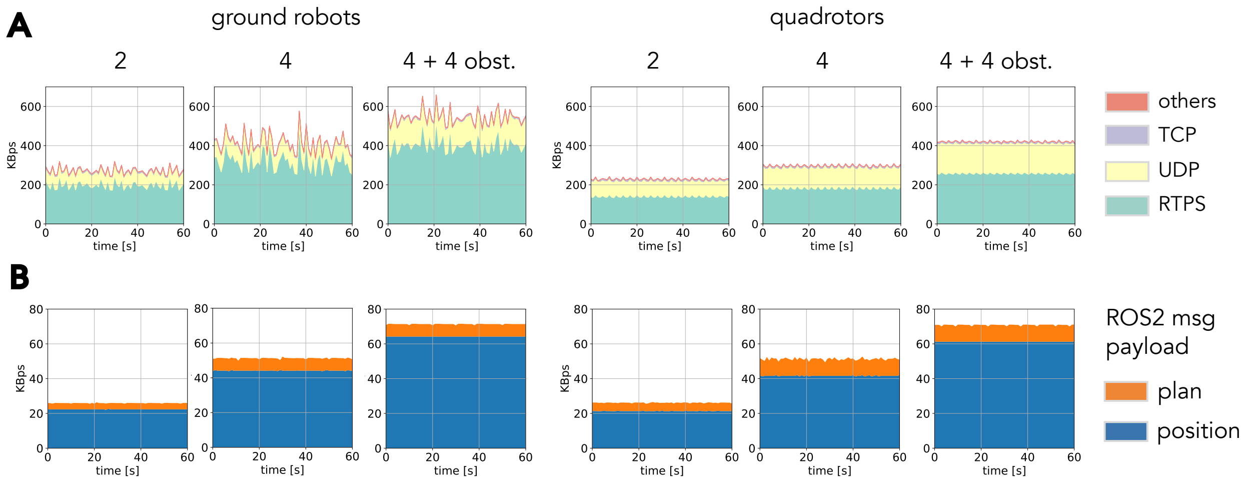

Figure S6: **Network usage breakdowns. (A)** We measure the amount of messages sent during missions in the lab, using Wireshark. The figures show breakdowns by message protocol. ROS 2 messages are with RTPS, while UDP is used for the motion capture system and quadrotor controls. **(B)** Further breakdowns of ROS 2 message payloads, generated from `ros2 bag` recording. The 'plan' category contains those used to distribute MAPF plans, and the 'position' category contains messages used for state estimation. Other message types use intra-process communication and do not add load to the router.

a time, which makes it difficult to benefit from using the GPU. Overall, both CPU and memory usage are moderate. The transformer inference is a one-shot operation and therefore difficult to see in CPU usage. Instead, the main consumer is MPC. Our choice of ACADOS [99] to solve MPC results in a computationally efficient implementation. These observations encourage us, although not currently within our scope, to allow other processes, such as perception, to run concurrently.

K Communication analysis

Our demonstration series builds on a reliable communications infrastructure, but in practice, especially in large deployments such as 40 robots, we find that packet loss is common and actually hinders aggressive quadrotor flight. Although this is not a limitation of the proposed method per se, we are interested in what is consuming the network, as well as the message delays for decentralized deployments, in order to establish further reliable setups.

K.1 Network usage

Figure S6(A) shows the breakdown of network usage in terms of packet protocol, for both ground robots and quadrotors, with and without obstacles. The main consumers are (i) RTPS, i.e., communication used in ROS 2, and (ii) UDP, which is used in the motion capture system and to send control commands to the quadrotors. Not reported qualitatively here, but further analysis reveals that the majority of UDP messages are for the motion capture system and that the use of the quadrotor is actually negligible. This explains why the amount of UDP messages is linear to the number of objects tracked, including obstacles. In terms of ROS 2 messaging, Figure S6(B) shows

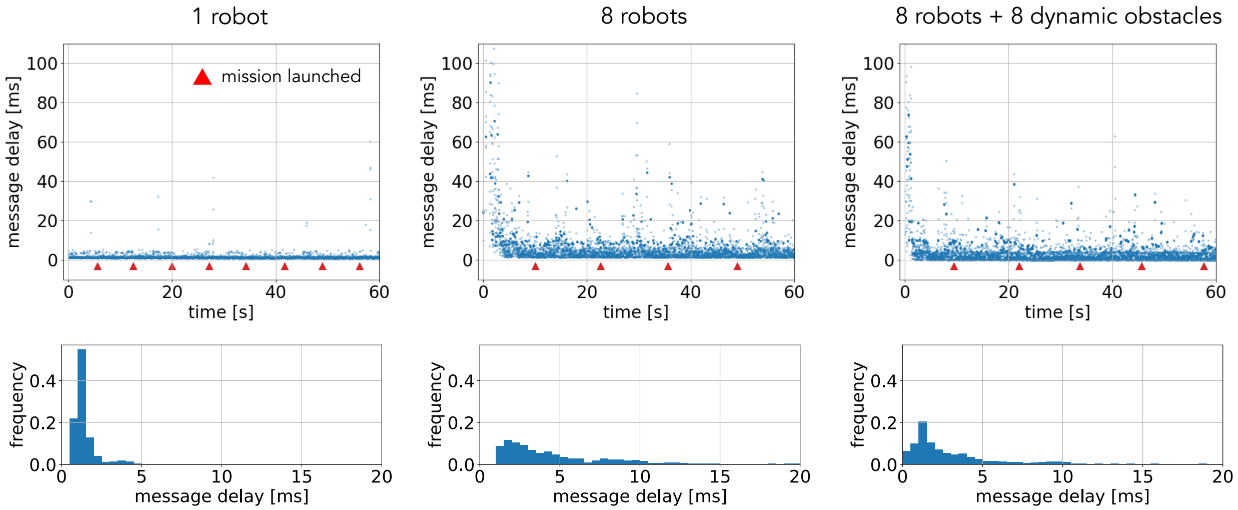

Figure S7: **Message delivery delay for onboard controllers in ground robots.** The data was collected with successive missions over one minute, using the same scenarios as in Figure 3. Each ROS 2 message is timestamped at the time of transmission, enabling us to estimate the message delay using the receiver clock. Prior to the experiment, the internal clocks of the sender and receiver are synchronized using Network Time Protocol. Robot-wise positional message is sent at 60 Hz, while the observation data is transmitted at 10 Hz. This different frequency takes into account that the size of the observation message can be huge, so sending it at a higher frequency will saturate the network capacity, which we experienced during the early development. Message delays are negligible, except for the first few seconds of launching ROS 2 nodes.

the message payload breakdown during missions, generated by `ros2 bag` recording. This reveals that the majority is again positional information for state estimation. Together with Figure S6(A), we conclude that the main communication load is to deliver state information from the motion capture system, not for distributing global plans to each robot.

Note that in Figure S6(A), the ground robot case could be noisy due to its decentralized deployments, while the quadrotor case is more stable due to its centralized deployments.

K.2 Message delively delay

We next measure the message delivery delay using ground robots. Figure S7 provides the measurement results, showing that the delay observed is tiny, even with eight robots deployed. This instant message delivery supports our demonstration of offloading state estimation for control synthesis from onboard computation.

L Delay tolerance for distributing global plans

The previous section reveals that the communication delay for decentralized deployments, while present, is almost negligible in our environment. In other environments, however, it can be difficult to implement a reliable, low-latency communication infrastructure. Theoretically, the framework's

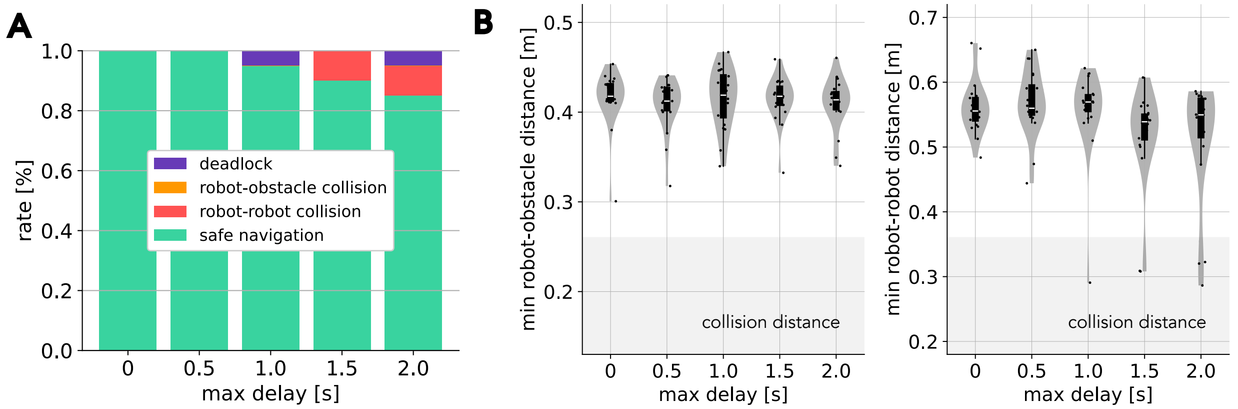

Figure S8: **Delay tolerance for global plan disassembly.** We deploy eight ground robots with eight obstacles, similar to Figures 4 and 7, but obstacles are *static*. In this experiment, each robot inserts artificial delays before starting its movements when receiving MAPF instructions. The delays are chosen uniformly at random within $[0, \text{max delay}]$ seconds, which are incoherent between robots. All robot-wise controllers are executed on the same machine as the central planner to eliminate the effect of communication delays. The figure shows **(A)** the breakdown of navigation results over 20 missions for each maximum delay and **(B)** the minimum robot-obstacle and robot-robot distances within each mission. The results clarify that the failures seen in Figure 7 are actually due to non-static environments with dynamic obstacles. In addition, the planner generates robust solutions for timing asynchrony to some extent.

reliance on communication is the distribution of central MAPF plans to each robot only.[2] We therefore evaluate communication delay tolerance when distributing MAPF plans by introducing artificial delays in message delivery.

We deploy eight ground robots with eight *static* obstacles. When receiving an MAPF plan, each robot artificially delays the start of the mission, with its period randomly sampled from $[0, \text{max delay}]$ seconds, which emulates the message delay. Figure S8(A) summarizes the breakdowns of 20 navigation tasks, with different maximum delay parameters. The result shows that without delays, the robots achieve zero failures, but as expected, we can observe failures as the delays increase. This is because MAPF plans are no longer feasible in terms of inter-robot collisions, as they assign spatiotemporal paths to each agent on the assumption that all robots start the mission simultaneously. As a result, together with the trajectory generation that drives the high-speed maneuver, we can see collisions between robots, even with the last-resort safeguards of CBFs. Still, the framework maintains a high success rate in these adversarial scenarios, owing to the robustness of MAPF-X solutions that explicitly address motion uncertainties.

Another insight from Figure S8(A) is that, from the zero delay condition, the failures observed in Figure 7(A) are caused by the presence of dynamic obstacles, which are not considered in MAPF-X. The adversary of dynamic obstacles is also evident from Figure S8(B), which shows that even in failure cases, there are no collisions with obstacles, unlike Figure 7(B). We discuss this aspect in detail in the next section.

[2]State estimation also uses communication from the motion capture system, but this is specific to our deployment setup and is not in the research subject of this paper.

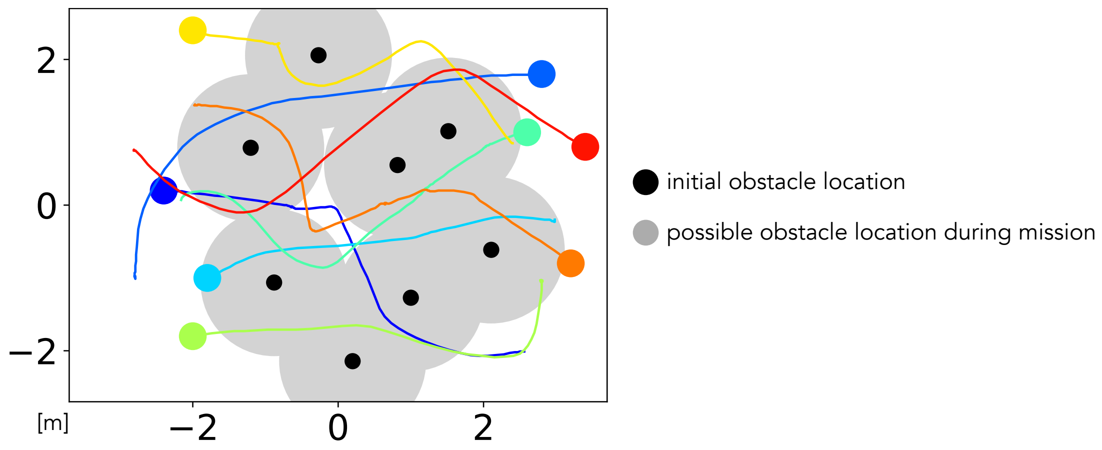

Figure S9: **Possible locations for dynamic obstacles.** Each dynamic obstacle moves slowly (5cm/s), but eight obstacles move continuously and unpredictably during missions. Consequently, the absolutely safe navigation areas for offline planning, colored white, are severely restricted, making it impossible to derive feasible solutions for such a conservative assumption. This forces the framework to perform planning such that intended trajectories potentially overlap with obstacle footprints, adapting online to dynamic environments. CBFs play a critical role in the online part. Meanwhile, this visualization also shows that mission failures are possible depending on configurations of dynamic obstacles, making the planning results completely infeasible.

M Presence of dynamic obstacles

For the ground robot deployments, Figure 7A with *dynamic* obstacles contains some navigation failures. Meanwhile, in Figure S8 with the same number of robots and *static* obstacles, we have not experienced such failures. This indicates that the presence of dynamic obstacles has caused unsafe execution. In practice, our framework has low sensitivity to environmental changes, i.e., allows for dynamic obstacles to some extent, thanks to the introduction of the online collision avoidance scheme. However, the increase in the number of dynamic obstacles may make the problem more difficult than our intuition suggests.

Let p be the probability that a robot trajectory, planned before it starts moving, will interfere with a dynamic obstacle during a mission. We say that an obstacle interferes with a trajectory if the robot's footprint derived from that trajectory overlaps with obstacles, causing the robot to deviate from the originally planned trajectory to avoid collisions. Suppose that the workspace contains M obstacles. Then the probability that a trajectory will not be interfered with by any obstacles is $(1-p)^M$. As we have N robots, the probability that all trajectories are not interfered by any obstacles is $q = ((1-p)^M)^N = (1-p)^{NM}$.

This q represents the probability that the offline planning can be executed without deviating from the intended trajectories. Let $M = 8$ and $N = 8$, like the ground robot deployments in Figure 7A. Reasoning p is difficult as it depends on the obstacle position, trajectory shape, and mission duration. However, even with very small p, q takes a non-negligible number, e.g., $p = 0.01$ leads $q = 0.53$. This explains why executing offline planning sometimes fails in the dynamic scenarios we tested, even with the slow obstacle speed of 5cm/s.

Figure S9 visualizes the regions that dynamic obstacles can reach during the ground robot's

mission. Obviously, conservative planning using regions where obstacles never reach is not feasible. This forces the robots to adapt to obstacle layouts online. As we have seen in the ablation study, CBFs adopted in the framework can play a crucial role in this. Meanwhile, depending on how the obstacles move during the mission, there is a risk that offline planning may no longer be feasible and some robots may end up in a deadlock situation, or, due to unexpected obstacles, the robots may have to deviate from their original trajectory at high speed, causing inter-robot collisions even with CBFs.

We emphasize that our intention to introduce dynamic obstacles is to demonstrate (i) real-time planning capability, as 'slow' planning would be completely obsolete in gradually changing environments, and (ii) low sensitivity of the framework to dynamic environments with online collision avoidance, (iii) while achieving kinodynamically aggressive maneuvers. This is different from designing safe, conservative navigation schemes with dynamic obstacles.

Supplementary Movies

Movie S1. Aggressive flights with drone swarm.

Movie S2. Lifelong operation with ground robots.

Movie S3. Deployment of 40 robots.

Movie S4. Last-mile delivery demonstration.

Movie S5. Warehouse automation demonstration.

Movie S6. Juxtaposing with decentralized drone control.
